# Glycomics and glycoproteomics of viruses: Mass spectrometry applications and insights toward structure–function relationships

**DOI:** 10.1002/mas.21629

**Published:** 2020-04-29

**Authors:** John F. Cipollo, Lisa M. Parsons

**Affiliations:** ^1^ Center for Biologics Evaluation and Research, Food and Drug Administration Silver Spring Maryland

**Keywords:** SWATH, MS^E^, CID, HCD, ETD, UVPD, glycopeptides, glycan, site occupancy, glycoinformatics, influenza, human immunodeficiency virus, vaccine, protein folding and stability, antigenic masking, lectins, receptor–ligand interactions, glycan array

## Abstract

The advancement of viral glycomics has paralleled that of the mass spectrometry glycomics toolbox. In some regard the glycoproteins studied have provided the impetus for this advancement. Viral proteins are often highly glycosylated, especially those targeted by the host immune system. Glycosylation tends to be dynamic over time as viruses propagate in host populations leading to increased number of and/or “movement” of glycosylation sites in response to the immune system and other pressures. This relationship can lead to highly glycosylated, difficult to analyze glycoproteins that challenge the capabilities of modern mass spectrometry. In this review, we briefly discuss five general areas where glycosylation is important in the viral niche and how mass spectrometry has been used to reveal key information regarding structure–function relationships between viral glycoproteins and host cells. We describe the recent past and current glycomics toolbox used in these analyses and give examples of how the requirement to analyze these complex glycoproteins has provided the incentive for some advances seen in glycomics mass spectrometry. A general overview of viral glycomics, special cases, mass spectrometry methods and work‐flows, informatics and complementary chemical techniques currently used are discussed. © 2020 The Authors. Mass Spectrometry Reviews published by John Wiley & Sons Ltd. Mass Spec Rev

## I INTRODUCTION

Viruses are similar to obligate intracellular parasites but lack the means for self‐reproduction outside a host cell and are generally not considered to be true living organisms. They are essentially parasites of the eukaryotic life style and capture the means of cellular reproduction. Among those systems commandeered by viruses are protein synthesis and co‐ and post‐translational modification (PTM). Glycosylation, as one of the most common PTMs, serves many functions, and viruses such as human immunodeficiency virus (HIV), influenza and others utilize this modification to advantage. Viral glycoproteins, like others, require glycosylation for proper folding, stability, and function. Areas that viruses can use glycosylation to advantage for specific functions include antigenic masking, interaction with innate immunity collectins, and receptor–ligand binding modulation. Other areas of importance extend into glycosylation's effect on viral vaccine safety and efficacy where glycosylation patterns produced by cell systems may impart qualities that affect these attributes. A brief description of these areas is summarized below to introduce the importance and essential nature of glycosylation in the viral niche.

## II MAJOR TYPES OF GLYCOSYLATION

Glycosylation is the most common form of protein PTM. About half of cellular proteins are decorated with some form of carbohydrate. The most common forms of this modification are N‐ and O‐linked glycosylation. Glycans perform many functions and effect biological processes in nearly every subdiscipline of biology and biomedicine. Glycosylation is involved in cell signaling (Cummings, 2019), cell–cell contact (Zanetta et al., 1994), innate immune response (York, Stevens, & Alymova, 2019), protein stability (Braakman & Bulleid, 2011), host pathogen recognition (Davicino et al., 2011) and many other areas (Varki, 1993). Glycosylation can be involved in a range of pathologic and pathogenic processes including metabolic diseases, cancer, and host–pathogen interactions (Gagneux AVaP, 2017). While it is not the focus of this review to describe glycosylation in detail, the most common forms, N‐ and O‐glycosylation will be covered in brief to introduce the reader to key elements of their biosynthesis and structure, which is important to better understand the analytics used in their study by mass spectrometry. This section covers mammalian, insect and plant cell glycosylation patterns. Cells derived from these families are used to propagate viruses and viral proteins. There are no viral glycosylation studies performed on patient derived virus and therefore little is known about the natural glycosylation profiles of viruses. All virus must be expanded in cell substrates. Therefore, the cell substrate determines the viral glycosylation pattern.

**Figure 1:**
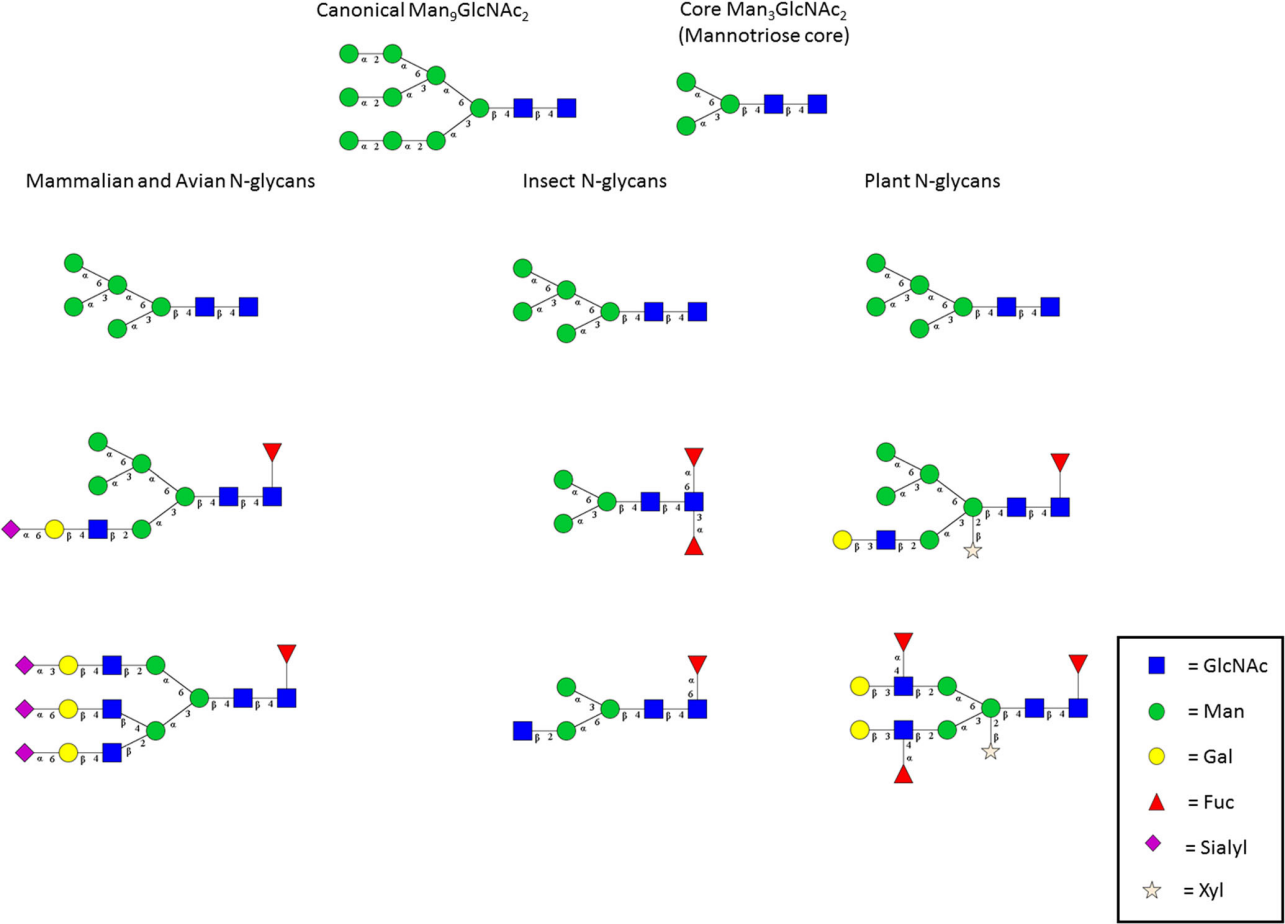
Major N‐glycans produced in cellular systems used in viral production. Top left is the canonical Man_9_GlcNAc_2_ glycan common to all eukaryotes except some protists. Top right is the canonical Man_3_GlcNAc_2_ seen in all N‐glycans. The far left column shows representatives of mammalian and avian N‐glycan sub‐classes: high mannose (top), hybrid (middle) and complex (bottom) types. The center column shows representatives of insect N‐glycan sub‐types: high mannose, paucimannose and abbreviated complex. The far‐right column shows representatives of plant N‐glycan subtypes: high mannose, hybrid, and complex. Plant and insect N‐glycans can contain Fucα1,3 linked to the core. Plant can contain Xylβ1,2 linked to the core Man residue. [Color figure can be viewed at wileyonlinelibrary.com]

N‐glycosylation begins with the synthesis of a canonical tetradecasaccharide of the composition Glc_3_Man_9_GlcNAc_2_ (Figure 1). It is transferred en‐block, co‐translationally, from its pyrophosphorodolichol intermediate to nascent protein regions by oligosaccharyltransferase (OST). The transfer occurs at regions containing the sequon NX(S/T), where X can be any amino acid except proline. Subsequent to transfer, endoplasmic reticulum (ER) and Golgi enzymes modify the saccharide by trimming in the ER and early Golgi stacks and then extending the remaining core, often composed of the core mannotriose linked to chitobiose (Fig. 1). In mammalian systems the resultant oligosaccharide is one of three subtypes: high mannose, complex, or hybrid; the last having structural features of the other two. The extensions most often contain GlcNAc, Gal, Fuc, and sialic acids (SAs) in a range of linkage configurations. Other cellular systems such as insects and plant, differ in overall N‐glycan composition, sub‐classes, and fine structure. Examples are shown in Figure 1. Common systems used in viral production are of mammalian, insect, plant cell origins (Minor et al., 2009). For a detailed review of these processes see (Pamela Stanley & Markus, 2017).

Important structural elements of glycans include composition, anomeric configuration, and glycosidic linkage. As polyols, saccharides possess a range of linkage points for the formation of glycosidic bonds, thus, the theoretical structural diversity is large. In reality, the structural range is limited genetically by the glycosylation‐active enzymes in a given genome (Moremen, Tiemeyer, & Nairn, 2012). For instance, insects tend to produce mostly short paucimannose (Man_3‐4_GlcNAc_2_) structures with minimal extension, although more complex N‐glycans have been reported and may serve transient functions or are less detectable due to low abundance in a minority of cells (Marchal et al., 2001; Shi & Jarvis, 2007). Plant systems produce extensions similar to mammalian N‐glycans; however, they also frequently contain core Xyl‐β1,4 substitution of the Man‐β1,4 core and also Fuc‐α1,3 substitution of the core GlcNAc residue closest to the aglycone (the sugar attached to the protein) (Kallolimath & Steinkellner, 2015). Both insect and plant derived cell lines are used as cell substrates for a range of licensed and proposed vaccines (Yusibov et al., 2006; Rodrigues et al., 2015). As Xyl and Fuc moieties can be safety concerns, analytical platforms should be in place to monitor production of such products. Insects may also produce methylated glycans and other sugar modifications that can be of concern when used in drug product manufacture. Representative N‐glycans of mammals, insects, and plants are shown in Figure 1. Importantly, a range of glycosidases and N‐glycopeptidases exist to remove N‐glycans to facilitate their study. While chemical means of release exist such as use of hydrazinolysis and other β‐elimination processes, the availability of enzymatic release has moved N‐glycosylation into the mainstream.

O‐glycosylation is also quite common. However, synthesis of this class of PTM is more complex. The majority of O‐glycosylation initiates with the transfer of GalNAc, via nucleotide sugar intermediate, to specific serines and threonines of the polypeptide backbone. Rather than a single OST, as in N‐glycosylation, multiple initiating polysaccharide GalNAc transferases (GalNAc‐Ts) are active in the process (Ten Hagen, Fritz, & Tabak, 2003). In mammalian systems, 24 overlapping polypeptide GalNAc‐Ts have been studied. Also, in mammalian systems, there are eight core types of O‐glycosylation referred to as cores 1‐8. These have been described in detail (Brockhausen, 1999). O‐glycosylation is far less well‐studied compared to N‐glycosylation primarily due to a dearth of enzymes available for release of O‐glycans across the range of core types. However, there have been some recent advances in this area and these shall be discussed later in Section V. Chemical release methods have been used for decades with β‐elimination remaining the standard for release and study of O‐glycans. However, chemical release methods do not leave behind peptide backbones that are easily analyzed for glycan position. Also, O‐glycosylation most often occurs in stretches of serine and threonine rich regions making positional analysis complex. Further complicating O‐glycosylation analysis, insect and plant O‐glycosylation is poorly understood making their analysis more difficult. For instance, *Drosophila* O‐glycans have unique core structures containing GlcA substitutions with some that initiate with fucosylation of the peptide backbone (Aoki et al., 2008). There is a need for better characterization for these cell substrates when viral antigens are proposed for use in vaccines or research, particularly those predicted to contain O‐glycosylation.

## III IMPORTANT FUNCTIONAL TARGETS OF GLYCOMICS ANALYSIS IN THE VIRAL NICHE

There are a range of viral function and fitness attributes related to protein glycosylation. Glycomics has been a powerful tool in revealing the chemical properties of glycans and their functions in the viral vaccine and host‐pathogen niche. Functions include protein folding and stabilization, antigenic masking or impact, interactions with the innate immune system, receptor–ligand interactions and vaccine safety and efficacy. A useful vantagepoint from which to view these targets of glycomics is briefly described below.

### A Protein Folding and Stability

Protein folding editing control is tightly linked to nascent glycosylation via OST activities and the associated ER and Golgi processes. Iterative folding events occur that are linked to the Parodi cycle: calnexin/calreticulin‐facilitated protein folding through oxidative iteration coupled to glycosidase II release of glucose from the nascent glycan (Parodi, 2000; Caramelo & Parodi, 2015). These activities have been shown to be critical in virus propagation (Gallagher et al., 1992; Hammond, Braakman, & Helenius, 1994). Indeed, inhibiting glycosylation‐dependent oxidative folding of the HIV envelope protein, gp120, in the ER impairs production of functional Env proteins (Walker et al., 1987). In influenza these events are linked to proper protein folding at hemagglutinin (HA) subtype H3 N‐glycosites N8 and N22. Loss of either site reduced efficiency of protein folding (Gallagher et al., 1988). Incomplete glycosylation at either of these sites may indicate inadequate protein folding. These two sites have historically provided insight into the mechanism of glycosylation by the OST enzyme complex (Hebert et al., 1997). They are close together on the nascent polypeptide backbone and are likely acted upon by both the STT3A and STT3B subunits of the OST complex, the latter of which is a “proof reading” subunit in the glycosylation process (Shrimal, Cherepanova, & Gilmore, 2015). The requirement for both N8 and N22 for proper folding and the nature of the OST complex makes this glycosylation event a possible drug target (Lopez‐Sambrooks et al., 2016; Puschnik et al., 2017; Baro et al., 2019). As viruses, especially retroviruses, propagate through the human population they tend to gain glycosylation sites over time, leading to more instances where glycosylation enzymes need to act efficiently. Therefore, it is likely that STT3A and STT3B functions become more important as viruses adapt to selective pressures leading to densely glycosylated protein regions. In this regard glycosite occupancy studies may reveal the necessity and efficiency of these OST functions.

### B Antigenic Masking

Viruses such as HIV and influenza gain and/or “move” glycosylation sites as they evolve in the human population. Protein regions targeted by the humoral immune system tend to exhibit high glycosylation density (Sun et al., 2012; Fang et al., 2014; Panico et al., 2016) leading to “glycoshielding” or “glycan masking” (Bragstad, Nielsen, & Fomsgaard, 2008; Lin et al., 2012), which refers to the reduced antibody response to protein antigenic sites in the proximity of glycosylation sites. By studying the rate of change of amino acids within antigenic sites, where a glycosylation site appears within a time domain, it can be revealed that in many cases antigenic drift can dramatically decreases within the antigenic site in the vicinity of the glycosylation site after it appears (Pentiah et al., 2015). The decrease in antigenic drift reflects reduced selective pressure for the virus to produce mutation and this is attributed to the function of the glycan to “shield” the region from immune pressure. While the majority of this work has focused on N‐glycosylation, glycoshielding has also been attributed to O‐glycosylation such as in HIV‐1 gp120 (Silver et al., 2020). As the density of glycosites increase so does the analytical challenge. Standard glycopeptide analysis using oxidoreduction and trypsin proteolysis coupled with Liquid chromatography–mass spectrometry (LC/MS) collision‐induced dissociation (CID) may not be adequate due to a high number of glycopeptides exhibiting multiple glycosites. To address these challenges, electron‐activated dissociation modalities such as electron capture dissociation, electronic excitation dissociation, electron transfer dissociation (ETD) have proven useful in the analysis of many highly glycosylated glycoproteins (Cooper, Hakansson, & Marshall, 2005; Wuhrer et al., 2007; Alley, Mechref, & Novotny, 2009; Wang et al., 2011a). Through a free radical cascade, odd‐electron species undergo rearrangement with subsequent cleavage of the N−Cα backbone bond (Zubarev et al., 2000; Leymarie, Costello, & O'Connor, 2003). Peptide fragmentation can take place inducing the formation of c and z• type fragment ions without loss of the glycan thus revealing its location on the peptide backbone (Elviri, 2012; Chen et al., 2018). While these electron‐activated techniques can be extremely useful, instruments capable of these experiments are expensive and not always available. Other strategies to address glycopeptides with multiple glycosites have involved the use of protease cocktails (MacCoss et al., 2002; Cao et al., 2018a). In such cases enzymes are chosen that complement one another in production of glycopeptides with only one glycosite. Instrumentation strategies will be discussed further in section IV.

### C Receptor–Ligand Interactions

Glycosylation sites may be present near, or as part of, a viral receptor‐binding domain (RBD). Such substitutions can affect binding properties such as affinity or ligand range. For instance, various influenza strains have gained glycosylation sites in the RBD (Jayaraman et al., 2012; Zhu et al., 2015). The significance of such substitutions is three‐fold. First, neutralizing antibodies tend to target the RBD and surrounding regions since they tend to be more effective at reducing infection (Xiu, Nakajima, & Nobusawa, 2008). Second, RBD specificities can change with amino acid or glycosylation change. A shift from zoonotic to human host can and has occurred through changes that result in a change in ligand specificity from Sialylα2,3 to Sialyαl2,6 substituted glycan chains (Lebarbenchon & Stallknecht, 2011). Therefore, due to the impactful nature of glycosylation associated with RBDs it can be imperative that the glycan composition/structure and occupancy at those sites be revealed. Third, ligands of viral receptors are often glycans. Characterization of the host glycan ligands targeted by individual viruses can be useful information in terms of pathogenicity, virulence, and tissue tropism. There is a tendency for the range of ligands bound by viruses to vary across virus clades and understanding these differences can offer drug targets and help to define population susceptibilities. Glycan arrays have been used in such efforts (Stevens et al., 2006b; Gulati et al., 2014; Tzarum et al., 2017) and this is especially so for HIV‐1, influenza, and parainfluenza. These endeavors can involve first characterization of host tissue glycans where infection occurs and then synthesizing or isolating the relevant compounds followed by characterization and printing onto arrays. The mammalian viruses that target glycan ligands have recently been reviewed and the list of those viruses is extensive (Thompson, de Vries, & Paulson, 2019). Eleven groups or families of viruses have been identified as targeting glycan ligands. These include: Adenoviridae, Polyomaviridae, Paraoviridae, Dependoparoviruses, Reoviridae, Caliciviridae, Coronaviridae, Picornaviridae, Arenavirus, Paramyxoviridae, and Orthomyxoviridae.

### D Interaction With Host Lectins

Host lectins (innate immune proteins that bind sugars) and collectins (collagen domain containing Ca^2+^‐dependent lectins) facilitate virus removal, modulate immune response, and are sometimes highjacked by viruses to enable entry into host cells. Examples include lung surfactant D (SP‐D), DC‐SIGN, dendritic cell mannose receptor (DC‐MR), Galectin‐1, and the Siglecs (Su, Gurney, & Lee, 2003; Hartshorn, 2010; Bermejo‐Jambrina et al., 2018; Wang et al., 2019; Chang & Nizet, 2020; Li et al., 2020). SP‐D, DC‐SIGN, and DC‐MR recognize high mannose glycans (DC‐SIGN can also recognize Fuc) on pathogen surfaces while Galectin‐1 recognizes β‐galactoside sugars. Lectins have been recently reviewed in the context of their role in viral defense and predation (Van Breedam et al., 2014). For instance, DC‐SIGN is predominantly expressed on dendritic cells and can bind and internalize HIV‐1 for degradation and promotion of major histocompatibility complex class I and II presentation but is also misused by viruses to access target cells (Moris et al., 2004, 2006). SP‐D, on the contrary, removes pathogens such as influenza from the lung via high mannose glycan recognition. Influenza gains glycosylation sited as it adapts to the human immune system in a yin‐yang relationship masking antigenic sites (Chen et al., 2019) but also gaining high mannose glycans which facilitate SP‐D‐dependent removal (Hsieh et al., 2018). The Siglecs are a large family of I‐type lectins with immunomodulatory function. Currently, there are 17 members of this SA‐binding cell surface receptor. They primarily recognize self‐associated molecular patterns (SAMP). They can be found on immune cells (Chang & Nizet, 2014; Laubli & Varki, 2020) such as monocytes and macrophages which have Siglec‐3, ‐5, ‐14 and ‐9. Siglec‐10 can be found on B cells. Siglecs‐3, ‐5, ‐7 and ‐9 have been reported to interact with HIV gp120 and recognition of this class of Siglecs may be involved in HIV‐1 attachment (Zou et al., 2011). The interplay between the Siglecs and glycans can have immunomodulary roles. However, abuse by viral pathogens does occur and is thought to have, at least in part, driven their rapid evolution to evade immune control (Laubli & Varki, 2020). Clearly, knowledge of glycan subtypes, particularly at specific glycosites in regions of interaction with lectins, can facilitate better understanding of these interactions, particularly in viral interactions with the host.

There is a growing body of literature providing evidence that O‐glycosylation may have a modulatory effect on the immune response (Madsen et al., 2012). A range of immune cell receptors have been shown to interact with O‐glycans. The major ones studied, along with their major recognized glycan, are macrophage galactose‐type C‐type lectins hMGL (Tn and Sialyl‐Tn antigens), mMGL1 (Lewis X), mMGL2 (Tn antigen, T antigen, and Core‐2), hSiglec‐3 (Sialyl‐Tn antigen), and hSiglec‐9 (Sialyl‐Tn antigen). These receptors, present on dendritic cells, can modulate immune response. In this way, depending on the O‐glycan encountered, the immune system may be pushed toward tolerance or be activated. The literature describing such interactions has been studied in the contest of cancer biology, which is beyond the scope of this review. However, a detailed review of current thinking in this area can be found here (Cornelissen & Van Vliet, 2016).

### E Vaccine Safety

The fine structure of glycans present in the natural infective state of viruses is not known as the ability to isolate virus from infected individuals in amounts large enough to study at current sensitivity levels is lacking. Instead, a range of cell substrates (cell lines, plant tissues, and hen eggs) are used for viral production for both research and vaccines with the assumption that such systems result in reasonably approximate glycoforms that are likely to occur in nature. Vaccine design and manufacture may or may not take the intended host's glycosylation into consideration. Currently, cell substrates used in viral vaccine production include embryonated hen eggs, *Spodoptera frugiperda* (sf9), Vero, Chinese hamster ovary (CHO), *Trichoplusia ni* (High‐Five), tobacco mosaic, and others (Oxford et al., 1987; Cox & Hollister, 2009; Minor et al., 2009; Zhang et al., 2012). Cell substrates used in vaccine production have been reviewed (Jordan & Sandig, 2014). Naturally, the glycosylation patterns of these cell lines will be reflected in the vaccine antigens produced and presented in the finished vaccine drug product. Some of these substrates may produce problematic glycan substitutions. Insect cells such as Tn (e.g., High Five) may produce Fucα1,3 substituted N‐glycan cores. These may be allergenic in some individuals. Similarly, plant‐based cell substrates not only produce Fucα1,3 but also Xylα1,2‐substituted N‐glycan cores, both of which may be allergenic in humans (Altmann, 2007; Seismann et al., 2010) (see Fig. 1). Essentially, all insect cells used for vaccine production synthesize primarily paucimannose glycans. These are short abbreviated glycan forms consisting mainly of canonical Man_3‐4_GlcNAc_2_ with or without core Fucα1,6 substitution. While these are not likely to be allergenic, they do present a high mannose substitution pattern which may differentially activate some immune system lectins and consequently downstream events that can influence response both positively or negatively (Turner & Hamvas, 2000; Atochina‐Vasserman, Beers, & Gow, 2010; Reichert et al., 2010). CHO cells produce Galα1,4 substitutions which can be allergenic and are the cause of alpha‐Gal syndrome (a food allergy to red meat) (Fischer, Yazdi, & Biedermann, 2016). In the context of vaccines, the ability to detect these glycan components is needed, particularly where cell substrates which could potentially produce allergens are used.

## IV INSTRUMENTATION AND FRAGMENTATION MODES: TARGETING THE DESIRED INFORMATION

Informative fragment intensities seen in glycopeptide data include those from peptidyl, glycosyl, core + peptide and oxonium ions. Peptidyl refers to those fragments that have lost one or more peptide bonds and facilitate peptide sequencing. Glycosyl, in this context, refers to fragments that retain the intact peptide but have lost a portion of the glycan through neutral loss. These are useful to verify glycan composition and may yield sequence data but rarely infer linkage configurations. Core + peptide refers to ions that have retained the intact peptide plus a portion of the N‐glycan core. These are often the most abundant ion intensities seen in CID‐based fragmentation spectra and have been used in informatic approaches as a gate keeper to identifying glycopeptide candidates for further processing (Dalpathado & Desaire, 2008). Oxonium ions represent portions of the glycan and are devoid of any peptidyl component. These aid in rationalizing the glycan moiety's composition and can also be used to identify glycopeptide spectra for further processing or during acquisition to target ions in subsequent scans. An optimal glycopeptide spectrum would contain abundant intensities from all four of these components. However, their generation depends heavily upon the instrumentation used, available fragmentation modalities, acquisition methods, and properties of the glycopeptides analyzed.

While data‐dependent acquisitions (DDA) remain popular, data‐independent acquisition (DIA) approaches such as SWATH and MS^E^ used in quadrupole time‐of‐flight (Q‐ToF) type instruments, have gained in popularity for glycoproteomics analyses. Some DDA methods may be triggered on the appearance of oxonium ion or core + peptide fragment ion intensities. The application of Orbitrap can utilize ion trap CID in combination with higher energy collisional dissociation (HCD) (Cruz et al., 2018). Experiments targeting glycopeptides with multiple glycosidic sites can be analyzed using ETD or other electron activated fragmentation modalities in efforts to retain the glycoside moiety whilst targeting the peptide backbone (Sarbu, Ghiulai, & Zamfir, 2014). In this way, the glycosites can be revealed along with their substituting glycan composition. ETD and HCD have been paired in a number of approaches in efforts to maximize abundance of all four fragment types listed previously and to identify the glycosite(s) with intact or nearly intact substituent composition due to ETD generation of c and z• ions (Saba et al., 2012; Singh et al., 2012). Ultraviolet photodissociation (UVPD), a relatively new mode of fragmentation, appears particularly capable of producing spectra rich in the four fragment types (Madsen et al., 2013; Ko & Brodbelt, 2015; Halim et al., 2018). All of these methods and related aspects will be discussed.

### A DDA Versus DIA

**Figure 2:**
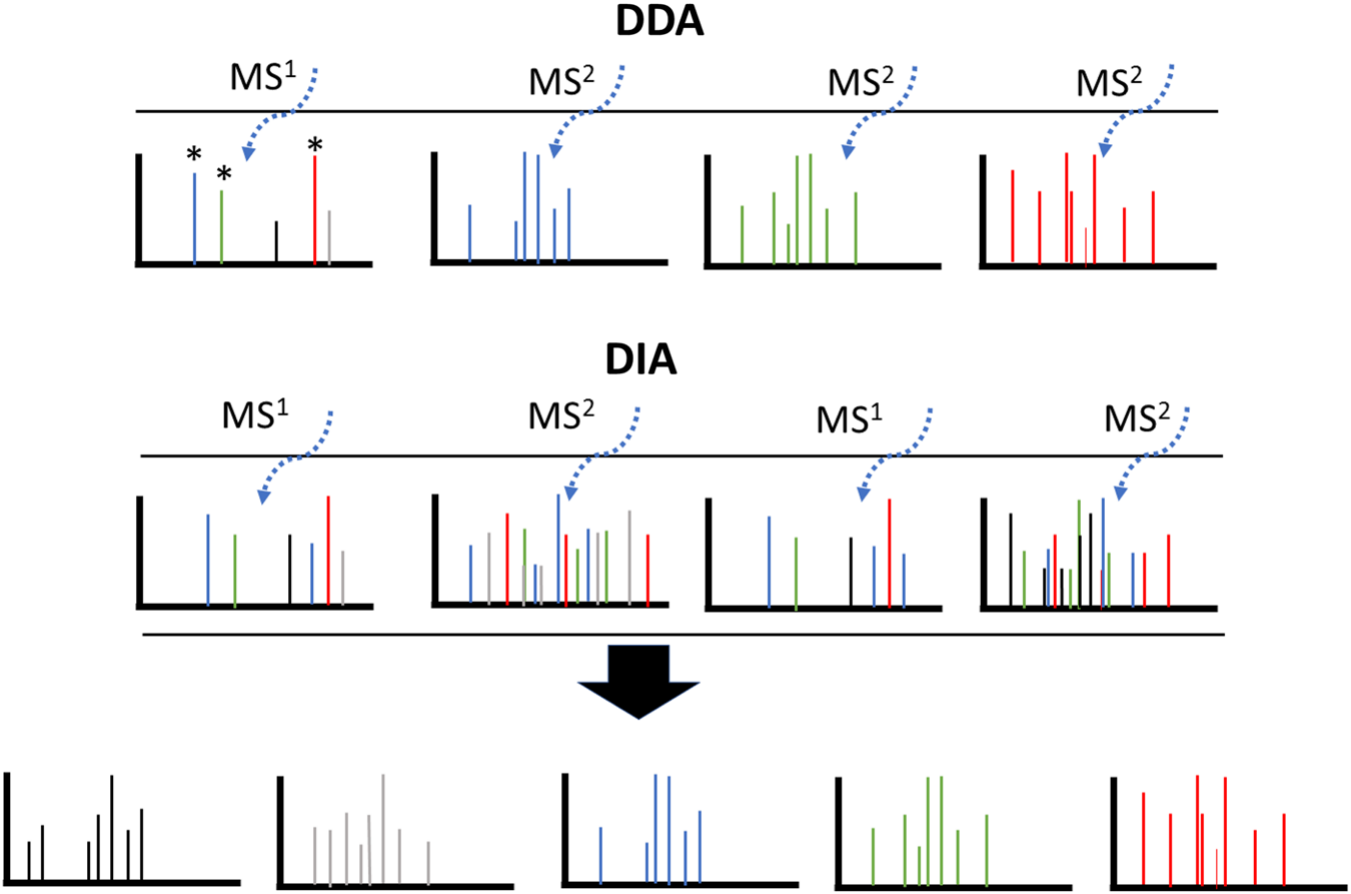
General differences between data‐dependent acquisitions (DDA) and data‐independent acquisitions (DIA). The top row represents a series of precursor ion scans within a small elution window where the same ions are seen (panel 1). Each precursor, demarked with a *, are targeted individually for a decomposition scan. Note that the fourth and fifth precursor ions are not selected for decomposition, for instance, because they were not in the target top three intensity list or not represented in an include list. The middle panel represents a DIA experiment. Two precursor ion scans are performed where a wider range of precursors are selected. The decomposition scans fragment multiple parent ions. Later these ions are deconvoluted informatically to yield more spectra and associated parent and daughter ions than the DDA method as represented in the bottom row. [Color figure can be viewed at wileyonlinelibrary.com]

The general scheme for DDA versus DIA data acquisition can be seen in Figure 2. DDA essentially targets single precursor ion *m/z* for decompositional analysis based on preformed criteria such as include lists or ion abundance. DDA remains a popular and relatively simple method of data acquisition when breadth and discovery are the main objectives; however, there are some disadvantages compared to DIA methods (Hu, Noble, & Wolf‐Yadlin, 2016). In LC/MS applications the sampling of such targets is stochastic and may not be adequate for quantitation. Additionally, unexpected ions may be missed if they are absent from the include list or DDA target strategy. While multiple rounds of DDA with added or alternative list members can, in part, alleviate some lost information, the method may not be discovery‐based ad initium. DIA methods, such as MS^E^ and SWATH circumvent both problems. Since sampling is more uniform, quantitation is more accurate. Potentially, all ions in the ion stream are analyzed and fragmented. Therefore, the DDA experiment is discovery based and this can be particularly useful for glycopeptide analysis where parent ions are densly packed in the elusion profile and represent multitude compounds that elute closely together but differ in fragmentation patterns. Methods such as SWATH and MS^E^, which key on fragmentation alignments, can capitalize on such densities leading to more identifications and these will be discussed further below.

### B SWATH and MS^E^

Sequential window acquisition of all theoretical mass spectra (SWATH) or Hyper Reaction Monitoring” (HRM), was first described by Gillet et al. (2012): This method leverages enhanced data acquisition via fragmentation of all ions in sequential and overlapping mass windows. It is systematic and reported to be non‐biased (Ludwig et al., 2018). Essentially, the strategy is based on peptide‐centric scoring, which relies on querying chromatographic and mass spectrometric coordinates of the proteins and peptides of interest in the form of peptide query parameters (PQP). The PQPs relate to peptide properties that are known prior to acquisition and relate to specific sample proteins and peptides, their elusion times, fragment intensities, etc. Recently developed algorithms can score data without empirically derived PQPs and may eventually replace the empirical forms (Teleman, Hauri, & Malmstrom, 2017). Thus, most published SWATH‐MS studies to date used spectral libraries generated by DDA experiments. The major advantage of SWATH‐MS is increased ion detection when compared to the traditional DDA experiment. While SWATH‐MS is not as quantitatively accurate as DDA it identifies more peptides and associated proteins and is more reproducible than traditional DDA (Bruderer et al., 2015; Kelstrup et al., 2018). While this technique has not been used in viral glycomics or glycoproteomics yet, we have included it here because of its increasing use in proteomics and recent introduction into glycoproteomic studies (Xu, Bailey, & Schulz, 2015; Zacchi & Schulz, 2016, 2019; Zhou & Schulz, 2020).

MS^E^ is similar to SWATH‐MS in that a low energy scan to collect precursor ions is executed prior to collection of fragment ions via a higher energy scan; however, both scans are continuous and not broken into overlapping mass windows. Truly independent of the data being collected, no ions are targeted, rather CID fragmentation is conducted on all ions passing through the T‐wave regions. Pseudo MS^2^ spectra are generated post‐collection using chromatographic elution properties of fragment and precursor ions (Geromanos et al., 2009; Li et al., 2009). Unlike SWATH‐MS, no file analogous to PQP parameters is required. The LC‐MS^E^ method is highly reliant upon the LC separation. Under typical data collection criteria if peaks are separated by one scan, or ~1.5 sec, the method can differentiate and accurately assign precursor and associate fragments (Geromanos et al., 2009). In practice, the method reaches its limit when glycopeptides elute closely and have overlapping fragment ions such as when the LC method resolving capacity is overwhelmed. Reverse phase C18 and hydrophilic interaction liquid chromatography (HILIC) chromatography systems are most commonly used. Reverse phase separations tend to elute glycopeptide families close together (Stavenhagen et al., 2017). In cases of close co‐elution the analyst must inspect neighboring spectra for possible mis‐assignment of fragment ions. HILIC elutes with more emphasis on the hydrophilic character of the glycopeptides (Yu et al., 2016) such that glycopeptides families tend to separate but overlap remains possible.

### C HCD

CID performed using an Orbitrap equipped with HCD, also known as higher‐energy C‐trap dissociation has become a popular mode of fragmentation for glycopeptides. HCD spectra generally contain fragment ion intensities of both the glycosyl and peptidyl moieties of the parent ion (Parker et al., 2013; Nilsson, 2016). Oxonium ion ratios of these spectra have been associated with the glycan composition and enantiomeric specificity. For instance, HexNAc‐Hex oxonium ions are characterized by the presence of associated daughter oxoniums at 126, 138, 144, and 168 daltons. For Galβ4GlcNAc‐terminated N‐glycopeptides, *m*/*z* 138 and 168 are intense with lower intensity of the 126 and 144 ions. Conversely, *m*/*z* 126 and 144 are relatively more intense than the *m*/*z* 138 and 168 ions for Galβ3GalNAc‐O‐ substituted peptide. Synthetic glycopeptides have been used to verify such assignments, and an informative (138 + 168)/(126 + 144) intensity ratio (GlcNAc/GalNAc ratio) can be used in these instances (Nilsson, 2016). Similar use of oxonium ion ratios has been applied in hybrid CID/ExD experiments as well (Scott et al., 2011; Yin et al., 2013). A relatively new HCD strategy is available on Orbitrap whereby stepped normalized collision energies (NCEs) are utilized. Glycan chain and peptide backbone informative fragment ions are generated (Yang, Yang, & Sun, 2018). Recent work has demonstrated that well‐tuned stepped energies have provided improved identifications art 1% false discover rates, site specific identifications with determinant fragment ions, and structure specific identifications with determinant fragment ions (Wang & Tian, 2020).

### D ETD Analyses and Variants

The utility of ETD was first exploited for glycomics analysis by McClucky and co‐workers (Hogan et al., 2005). Electron‐activated fragmentation dissociation modalities operate through a free radical cascade induced by transfer or capture of odd‐order electrons. The approach, when optimized, preferentially targets the N–Cα bond of the peptide backbone to produce c ions and z^•^ radical ions. Fragmentation of the substituting glycans is minimal. The intact glycan is often retained and its location in the peptide can be derived. This can be particularly useful in glycopeptides where more than one glycosite is present as is often seen in viral proteins such as seasonal influenza HA and HIV Env. The glycan subtype can be revealed based on composition of the still intact glycan. Along with position, knowledge of the subtype allows inference of the potential impact of each glycosylation site to biological activities such as lectin interactions. Examples will be presented later.

The efficiency of fragmentation was improved by Coon and co‐workers with the development of EtCaD or electron transfer/collisionally activated dissociation (Swaney et al., 2007). This was implemented to overcome the predominance of doubly charged ion fragmentation efficiency by ETD. The NCE and Q‐activation values are lowered from typical CAD conditions and the ions are first subjected to ETD dissociation followed by CAD excitation. The result is an increase in fragmentation efficiency. Another hybrid experiment, a combination of ETD and HCD as coined by Thermo, has been applied frequently to glycopeptide analysis. This experiment combines HCD with ETD. The ions are excited, for example, in an ion routing multipole or C‐trap and then subjected to ETD in a linear trap. This electron transfer higher energy collision dissociation (ETHCD) experiment yields greater fragmentation of the N–Cα bond yielding abundant C and Z^•^ ions. Oxonium ions are produced during the HCD segments and can be used to target the ETHCD experiment specifically to glycopeptide analytes (Hu et al., 2016). The Coon group also recently developed the AI ETD method. A Thermo Fusion Lumos was modified with a CO_2_ laser for this application. The method uses concurrent infrared (IR) photoactivation to improve dissociation efficiencies and product ion generation in ETD experiments (Riley et al., 2019). Resulting spectra are rich in (1) unmodified peptide backbone fragments (i.e., b/y/c/z‐type); (2) peptide backbone fragments with intact glycan still attached; (3) peptide backbone fragments with only a HexNAc moiety still attached; (4) Y‐type ions (intact peptide plus glycan fragments referred to elsewhere herein as glycosyl fragments), and (5) oxonium ions. While instrumentation to perform this experiment is not currently widely available the technique appears promising.

### E UVPD MS Analysis of Glycopeptides

UVPD has not yet been used in viral glycomics applications. However, this fragmentation method has been recently reviewed (R Julian, 2017) and shows promise in glycoproteomics (Madsen et al., 2013; Ko & Brodbelt, 2015). UVPD has been used successfully in top down, middle‐down, and bottom up proteomics approaches. The high energy deposition and wavelength options becoming available for UV photoactivation can be utilized to achieve efficient fragmentation resulting in high sequence coverage. UVPD has been carried out over a range of wavelengths, including 266, 213, 193, and 157 nm. At 266 nm (Park et al., 2009; Yeh et al., 2009) absorption occurs primarily at tyrosine and tryptophan sidechains in proteins. At higher energy wavelengths of 193 nm (Moon, Yoon, & Kim, 2005; Morgan, Hettick, & Russell, 2005) and 213 nm (Moon, Yoon, & Kim, 2005; Morgan, Hettick, & Russell, 2005), excitation of the peptide backbone occurs. Wavelength excitation at 157 nm (Thompson, Cui, & Reilly, 2004) can be used for more broad bond coverage. Once an excited state electron is generated, two possible relaxation modes are available. One happens on the femtosecond time frame and the electron moves into a dissociative orbital leading to a prompt dissociation. In this case, there is no energy redistribution. This could be an advantage for glycopeptide analysis as rearrangements would be less likely to occur such as those seen in fucosylated forms (Wuhrer, Deelder, & van der Burgt, 2011). The second relaxation involves internal conversion of energy into vibrational modes resulting in intramolecular vibrational energy redistribution (IVR) (Wuhrer, Deelder, van der Burgt, 2011). The second relaxation involves internal conversion of energy into vibrational modes. The dictates of intramolecular IVR (Stannard & Gelbart, 1981) enable this vibrational energy to redistribute among all available modes within the femtosecond to picosecond timescale. The result is a vibrationally hot molecule and, if the photon energy is sufficient to cause dissociation via internal conversion, the fragments produced will be similar to those generated by CID. The two modes, direct dissociation and internal conversion, work together to provide the efficient fragmentation seen with this mode of dissociation.

Byoung et al. (Ko & Brodbelt, 2015) compared glycopeptide CID and UVPD at 193 nm in a linear trap. UVPD on deprotonated (negative polarity mode) glycopeptides yielded spectra rich in both glycocidic and peptide bond cleavages with unique fragments from formation of Y‐ions due to cleavage at the N‐terminus of proline. In positive polarity mode with protonated glycopeptides, CID and UVPD produced primarily fragment ions from glycan cleavages. Alternatively, Reilly and coworker (Zhang & Reilly, 2009) performed UVPD at 157 nm and obtained both glycan and peptide sequence information in spectra that was rich in x‐, v‐, w‐, and y‐type peptide fragment ions that retained the glycan, as well as spectra where both glycosyl fragments and cross‐ring cleavage products were observed from N‐linked glycopeptides.

Wavelength choice can dictate the information content of the resultant spectra. At 266 nm the peptide the backbone is not effectively cleaved. At 213 and 193 nm these bonds are more efficiently cleaved due to absorption of energy in the peptide bond. The 157 nm wavelength targets a broader range of bonds and favors short timescale dissociation. UVPD use in glycopeptide and released glycan analysis is still in an early stage. Both positive and negative polarity modes have shown promise. Fragmentation patterns appear to be highly informative and could prove to be a useful addition to the collective glycomics analysis toolbox.

## V GLYCOINFORMATICS: CURRENT TRENDS AND PROJECTIONS

We have surveyed the most recent literature for software and instrumentation used in viral glycoproteomics workflows. Eighteen glycoproteomics publications are reviewed. Data processing strategies including informatics are highlighted. Instruments used included Thermo linear trap quadrupole fourier‐transform ion cyclotron resonance (LTQ FT‐ICR), Thermo Orbitrap mass spectrometers, and Waters Synapt G2 series instruments. The Thermo and Waters instruments collected data using DDA and DIA respectively. Ionization types included CID, HCD, and ETD. The informatics used in these recent studies included GlycoPeptide Search (Pompach et al., 2012), Protein Metrics (Bern et al., 2012) software Byonic™ (and Byologic™) (Bern et al., 2012), BiopharmaLynx™ from Waters, the ProLuCID algorithm from Integrated Proteomics Pipline‐IP2 (Eng, McCormack, & Yates, 1994), and a combinations of manual assignment of glycan moiety assisted with GlycoMod (Cooper, Gasteiger, & Packer, 2001) or Biopharm Finder (Thermo Fisher Scientific) and protein assignment using Mascot.

GlycoMod, an online program used to predict glycan compositions from mass, was published in 2001. Since then the number of hardware and software options available to collect, interpret and assign glycan and glycopeptide mass spectra has blossomed. Some are instrument and data type specific, such as BiopharmaLynx™ from Waters, while others can interpret mzXML or mzML files from multiple instruments such as GlycoPeptide Search or Byonic™. Optimally, for intact glycopeptide analysis, the chosen software should be capable of assigning both peptide and glycan fragment ion intensities from both N‐ and O‐glycans. Other considerations include the: range of proteases and other decompositional applications made available in the software, software capacity for protein sequence input (typically not a problem for viral glycomics), and type of peptide and glycan databases available. The approach for spectral assignment and scoring can be dependent upon the data type collected. For example, some programs use oxonium ions as filters to identify glycopeptide spectra (Pompach et al., 2012; Lynn et al., 2015; Toghi Eshghi et al., 2015) while others use them for scoring (Bern et al., 2012; Woodin et al., 2012). This may work well for MS^2^ data, but may not for MS^E^ or SWATH, since there can be a small amount of overlap between neighboring spectra. Hybrid and combination fragmentation schema are also considerations in the informatics coding as the different fragmentation modalities can produce characteristic fragmentation patterns. Thus, such hybrid experiments may require specific informatics treatment. Strategies for comparison of multiple injections may also be desired and a number of approaches have been employed. A range of software have been used in viral glycomics study and these have been covered in recent reviews which cover up to 2017 (Dallas et al., 2013; Tsai & Chen, 2017; Narimatsu et al., 2018). Here we cover more recent studies and their associated informatics.

A range of reports used the Protein Metrics (Bern et al., 2012) software Byonic™ (and Byologic™) to process data collected on Orbitrap instruments. Byonic is a commercially available software that can process MS^n^, SWATH, and MS^E^ data. Standard file formats are supported (MGF, mzML, mzXML, or Thermo RAW). The software can identify both N‐ and O‐glycosylation, uses glycan databases of known or user supplied glycans, and can accept a large protein database. It uses core (peptide + core glycan components) and oxonium ion fragments for scoring and can identify both peptidyl and glycosyl fragments. Struwe et al. (2018), and (Behrens et al., 2017, 2018; Sarkar et al., 2018; Struwe et al., 2018), performed studies on the HIV envelope protein. HCD fragmentation was used on glycopeptides to obtain spectra containing predominantly glycosyl fragments (Behrens et al., 2016). Their workflows included LC/MS^E^ with ion mobility for analysis of free‐glycan and the resultant glycan identifications were used to construct a glycan library for glycopeptide assignment. The software used to assign the free‐glycan fragmentation was not specified. Urbanowicz et al. (2019) used HCD fragmentation with Byonic processing to characterize the Hepatitis C E2 envelope protein. Cruz et al. (2018) used a combination of CID (for glycosyl ions) and HCD (for peptidyl ions) to analyze the glycosylation of flu H1N1 HA. HCD data was processed by the vendor software Proteome Discoverer™ 2.0 and then analyzed by Byonic. Diagnostic oxoniums were manually verified in the HCD data. CID data was manually assigned. Pegg, Hoogland, & Gorman (2017) used a HCD‐product‐dependent (pd)‐ETD or ‐CID strategy to identify both N‐ and O‐glycopeptides of the Newcastle Disease Virus (NVD) HA‐neuraminidase (NA) protein. Oxonium ions produced by HCD were used to trigger either ETD or CID. For each sample three separate chromatographic experiments were performed: one with HCD only, one with HCD‐ETD, and one with HCD‐CID. Proteome Discoverer and the Mascot search engines were used to search for peptides in the HCD MS/MS data using a chicken protein database as well as an NVD database. Peptides not assigned were sequenced *de novo* using the Xcalibur Qual Browser (Thermo Fisher Scientific). An in‐house program called OxoExtract was used to search the HCD MS^2^ analysis for oxonium ions, core glycosyl ions, and to query GlycoMod for prediction of the glycan composition. OxoExtract compositions were used to create a custom glycan database to search the CID and ETD data with Byonic. The identified glycopeptides were manually validated in the Xcalibur Qual Browser.

Four papers reviewed used the vendor software, BiopharmaLynx™, to process MS^E^ data acquired on a Synapt G2 from Waters. BiopharmaLynx (BPL) processes LC/MS^E^ spectra through LC‐dependent alignment of fragment ion scans with their respective precursor ion scans, deisotopes, and deconvolutes for final spectral presentation. It assigns glycopeptides primarily through assignment of peptidyl but not glycosyl, oxonium or core + peptide fragments. The software can accommodate many peptide modifications but works best with one to a few protein sequences. BPL also allows the user to compare two injections. Protein sequences and glycan compositions are input manually. In addition to BPL, Ivleva et al. (2019) used Water's MassLynx for manual inspection and Water's MaxEnt3 for peptide *de novo* sequencing to study the HIV Env protein. Because BPL only assigns glycopeptides based on the whole mass of the glycan, the authors assigned the glycosyl and oxonium ion fragments manually to verify the automated assignments were correct. They found eleven automated assignments that were incorrect due to near coincident masses arising from peptide–glycan combinations. An et al. (2019) also used manual interpretation to verify the BPL assignments of flu HA were correct. Parsons et al. (2017, 2019) studied flu HA and the spike protein from infectious bronchitis virus. The in‐house program GLYMPS was used. The software automatically assigns glycosyl, peptidyl, oxonium, and core + peptide components of the spectra. GLYMPS allows comparison between all processed injections data. BPL must be used to first process data.

Cao et al. (2017, 2018b) took a simplification approach to site‐specific glycopeptide characterization. The glycopeptides of HIV‐Env were digested first with Endo H followed by PNGase F to leave a 203, 3 (incorporation of ^18^O), or 0 amu modification indicating high mannose/hybrid, complex, or no occupancy at each site. They also used several different proteases to maximize sequence coverage. These minimal modifications eliminated the need for a glycan library and for software capable of assigning glycan fragments. Instead, they used the ProLuCID algorithm from Integrated Proteomics Pipline‐IP2 (Eng, McCormack, & Yates, 1994) to search the CID MS/MS data and DTASelect (Tabb, McDonald, & Yates, 2002) to filter the results for quality and to include only peptides with potential glycosylation sites. This approach can predict glycan subclasses but information concerning the true composition of the original glycan moieties is lost.

Two reports used an MS^2^ targeted strategy. CID MS^2^ was used to generate glycosyl fragments followed by MS^3^ targeting of core + peptide fragment ions to generate peptidyl fragments. She et al. (2017) and Liu et al. (2017) analyzed influenza proteins. She and co‐workers used an LTQ FT‐ICR and Liu et al. used an LTQ‐Orbitrap XL. Peptides were identified with Mascot (Matrix Science, London, UK) and the NCBI nonredundant influenzae protein database and an in‐house influenza database was used. Oxonium ions were used to identify glycopeptide spectra and the glycosyl fragments were assigned manually. Liu et al. used Biopharm Finder (Thermo) to identify glycosylated peptides, then assigned the MS^2^ and MS^3^ manually.

Smargiasso et al. (2019) studied human cytomegalovirus glycoprotein B grown in different cell lines. They used an in‐house program to search MS^2^ HCD data for oxonium and HexNAc + peptide ions, then manually curated the glycosyl‐containing MS^2^ spectra. Glycan composition was determined from the precursor mass using GlycoMod. A Mascot search engine was used to assign de‐glycosylated peptides.

Nordén et al. (2019) used HCD and ETD to study the N‐ and O‐glycosylation of recombinant glycoprotein E (gE) from Varicella Zoster Virus. Precursors with the highest charge states and most intense ions triggered MS^2^ HCD. Oxonium ions present in the first HCD scan triggered a second MS^2^ scan: either ETD or HCD at a higher energy. An in‐house Mascot server was used to search the data for peptides from the gE protein sequence, four possible core glycans in both HCD and ETD, and oxonium ions in HCD. Annotation of the glycosyl fragments and assignment of the mass difference between the peptide and the precursor masses is assumed to be manual as no software was listed.

Zhu et al. (2019) used oxonium‐ion triggered HCD at three different energy levels to study recombinant H1N1 flu NA. Deglycosylated peptides were assigned using Proteome Discoverer 2.1 and MaxQuant using the NA sequence of the virus. Files were converted to mzML format using the Trans‐Proteome Pipeline (Deutsch et al., 2010). GPQuest (Toghi Eshghi et al., 2015) searched the mzML files using a glycopeptide database consisting of the NA peptides with known glycans from the cell line used. GPQuest is free software that uses oxonium ions to filter HCD data then assigns precursor and core glycolyl fragments of N‐ and O‐glycosylated peptides.

Of all these studies, only three reported submission of their data in a public repository. Cao et al. (2017, 2018b) deposited their data in Mass Spectrometry Interactive Virtual Environment (MassIVE) (Spectrometry CfCM) while (She et al., 2017) deposited theirs in PRIDE (Vizcaíno et al., 2015). PRIDE and MassIVE both focus on supporting tandem MS. PRIDE allows users to upload either “partial” or “complete” datasets with both sets containing raw data and standard metadata. The difference is that complete datasets have been annotated and assigned in a supported open data format whereas partial datasets have output files in other formats (Vizcaíno et al., 2015). MassIVE is a web server used to analyze MS/MS data online. Its database can be searched by peptide sequence, variant peptide sequence, post‐translational modifications (i.e., with a UNIMOD code), and protein name. Other databases include the Global Proteome Machine Database (Craig, Cortens, & Beavis, 2004), Chorus (Amazon Web Services I, 2013), and The MaxQuant DataBase (MaxQB) (Schaab et al., 2012), and have been reviewed by Perez‐Riverol et al. (2015).

Three observations can be made from this abbreviated survey. First, there seems to be a preference to assign glycopeptides spectra with commercial software, in‐house software, or manually. Only Zhu and co‐workers used free software (GPQuest) to assign LC/MS data. Second, many of the workflows involved multiple software either for data conversion or to search and assign the peptidyl and glycolyl moieties separately. Third, few researchers deposit their data in public databases. This is understandable as most databases are peptide‐centric and do not address all of the concerns that come with glycoproteomics data, particularly those collected and processed in unique ways. A new option for glycoproteomic data is GlycoPOST (Center NBD, 2018) which allows users to submit raw data and peak lists. Associated experimental information in GlycoPOST is based on the Minimum Information Required for a Glycomics Experiment guidelines (Kolarich, Rapp, & Struwe, 2013; Campbell et al., 2019). As more journals such as *Nature* (Flannery et al., 1992 *)*, *Proteomics* (Sandy et al., 1991), and *Molecular & Cellular Proteomics* (Kainulainen et al., 1998 *)* require proteomic or raw mass spectrometry data be deposited in public databases, proper deposition of data will be an important consideration. More information on standardization of bioinformatics of N‐ and O‐glycosylation can be found in the recent review by Rojas‐Macias et al. (2019).

## VI MASS SPECTROMETRY PATHWAYS TO MEANINGFUL GLYCOMICS DATA

**Figure 3:**
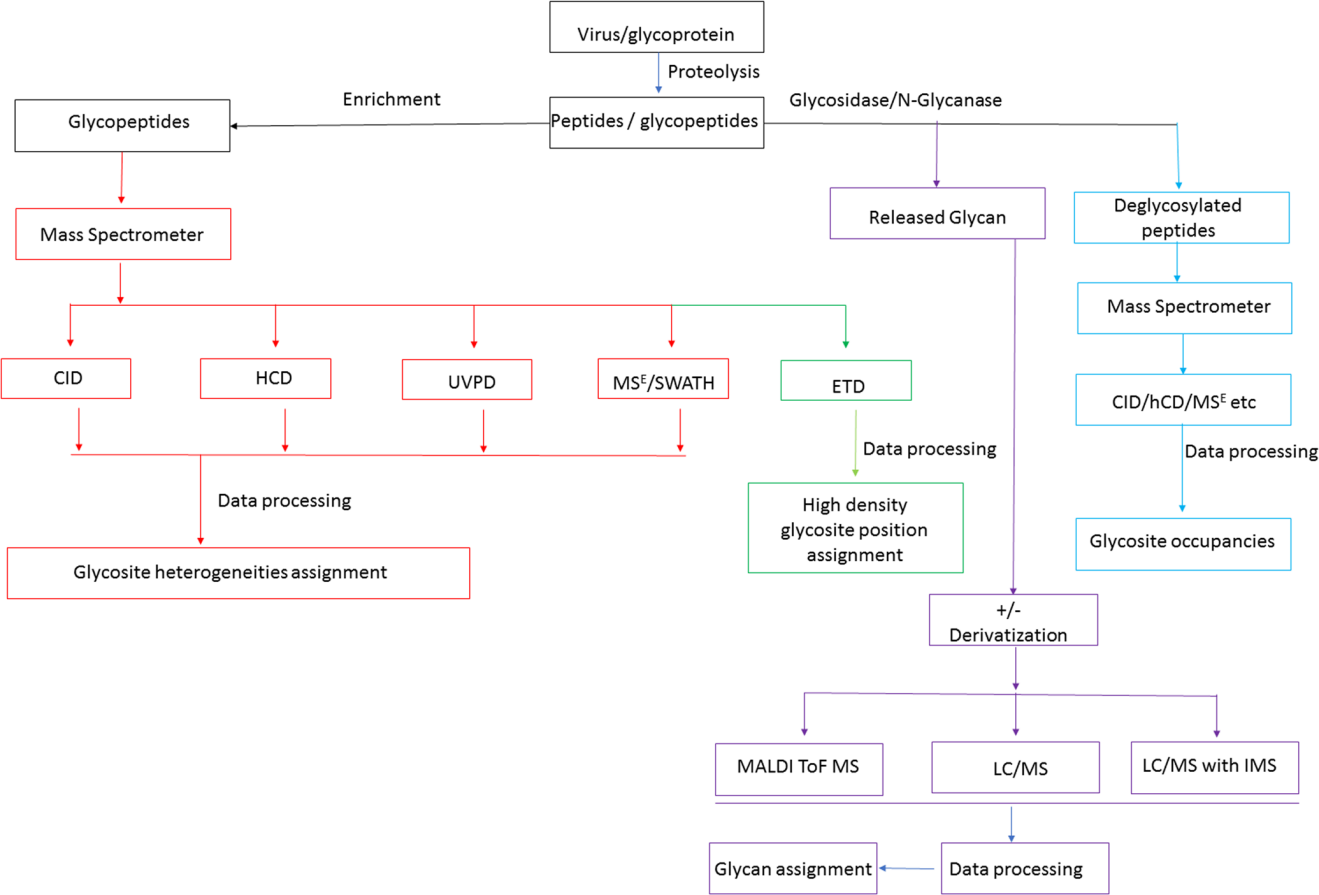
General glycomics workflow for virus analysis with multiple options shown. The four major pathways lead to analyses of glycosite heterogeneities assignment (red), released glycans (purple), deglycosylated peptides for site occupancy estimates (light blue), and high glycosite density position assignment (green). Glycopeptides can be enriched using a range of processes. They can be analyzed using different fragmentation techniques such as collision‐induced dissociation (CID), higher energy collisional dissociation (HCD), ultraviolet photodissociation (UVPD), electron transfer dissociation (ETD) and combinations thereof as well as data acquisition methods such as MS^E^ and SWATH. Each of these modes of analysis provides spectra with characteristic information content as discussed in the text. Released glycans can also be enriched prior to analysis in native or derivative forms. Three major analytical methods used are shown in the figure: matrix‐assisted laser desorption/ionization‐time‐of‐flight mass spectrometry (MALDI‐ToF MS), liquid chromatography–mass spectrometry (LC/MS), and LC/MS with ion mobility. Deglycosylated peptides are analyzed in strategies to estimate glycosylation site occupancy. Common fragmentation strategies used are HCD, CID and MS^E^.

The glycomics analytical strategy for viral glycoprotein antigens depends on the question(s) asked and the required level of structural information to address these questions. The current comprehensive analytical work‐flow analyzes released glycans for overall glycosylation, intact glycopeptides for site specific heterogeneity, and glycopeptides that have been treated to release their glycan moiety to study site occupancy. It is generally accepted that glycan structural elements are best revealed from released glycans as derivatization processes can facilitate analysis without the complication of peptide modification and degradation. Desired information for the released glycans can include composition, glycosidic linkage configuration, overall abundance of each glycoform, and branch structure. Intact glycopeptide information can include site of occupancy, composition, and salient features of the glycans such as inferred branch structure and linkage, and site occupancy percent. Inferred structural assignments should be made with caution for various reasons not least of which is the possibility of intramolecular migration (Wuhrer, Deelder, & van der Burgt, 2011). A generalized work‐flow encompassing the major analytical options that can be used can be found in Figure 3.

It is impotent to be aware of the cell system and resultant matrix used to generate the sample as some can employ animal‐based serum or factors which can contaminate the viral preparation, complicating downstream analysis especially for released glycans. Alternatively, virus protein samples may be generated by recombinant technologies yielding highly enriched or purified protein or antigen. In both cases it is important to choose a cell substrate that reflects the biological context being investigated such that any glycosylation is reflective of the intended query. For instance, HEK293 cells may be used to produce influenza HA. However, these cell lines can produce abundant highly complex and sialylated glycans (Gugliotta et al., 2017). Native influenza virus HA is completely or nearly completely devoid of SA through the activity of virus NA and desialylation is a requirement for some properties of viral production, host cell recognition and invasion. Therefore, any conclusions made from highly sialylated HA should be make with caution.

As shown in the generalized workflow in Figure 3, the virus or expressed protein is proteolyzed and the hydrolysate is split into two aliquots. One aliquot (to the right) is treated with releasing glycosidase or glycosylamidase and the glycans and deglycosylated peptides are collected separately. The second aliquot, containing intact glycopeptides (left), can be subjected to glycopeptide enrichment via HILIC, porous graphitized carbon, lectin affinity, size exclusion chromatography or other selective process (Calvano, Zambonin, & Jensen, 2008; Wohlgemuth et al., 2010; Wang, Wu, & Khoo, 2011; Zhang et al., 2012). This step, while not required, can be used to decrease content of the more highly ionizable peptides and other contaminating compounds allowing for improved detection of the hydrophilic and relatively poorly ionizable glycopeptides (An & Cipollo, 2011).

### A Released Glycan Analysis

Released glycans can be derivatized prior to analysis using a range of approaches (Ruhaak et al., 2010). A popular and well described derivatization approach uses permethylation (Viseux, de Hoffmann, & Domon, 1997; Viseux, Costello, & Domon, 1999) and LC/MS methods have been developed to facilitate its use (Novotny & Mechref, 2005; Costello, Contado‐Miller, & Cipollo, 2007; Müller, 2009). Prior to permethylation, the glycans can be reduced to prevent separation of the reducing end anomers. During permethylation, protons on hydroxyl, amine, and carboxyl groups are exchanged for methyl groups, making the analytes more hydrophobic and size neighbors more equivalent in the mass spectrometer, thus increasing sensitivity and rendering the oligosaccharides in biologically related samples more representative of actual relative abundance. The per‐O‐methylation pattern yields mass tag information upon fragmentation that is useful for branch point identification as well as identification of fragments arising through cross ring fragmentation (Reinhold, Reinhold, & Costello, 1995; Solouki et al., 1998; Wuhrer & Deelder, 2006). Permethylation renders glycosidic and cross ring bond fragmentations in similar energy ranges yielding significant ion abundances of both types. Thus linkage, sequence, and branch information can be obtained from tandem MS analysis using, for instance, ToF/ToF or other analyzer to generate MS^2^ or higher MS^n^ for decompositional analysis. If only composition is required, MALDI‐ToF MS is sufficient and can be performed semi‐quantitatively (Viseux et al., 2001). Tandem MS can be performed to potentially yield nearly complete structural assignment except for anomeric configuration and enantiomer identity in cases where it cannot be inferred.

An alternative approach is to derivatize the reducing end with labels such as 2‐aminobenzamide (2‐AB) or 2‐aminobenzoic acid (2‐AA) prior to analysis by LC/MS or MALDI ToF MS. Positive ion mode CID fragmentation, for instance, with hydrogen‐adducted glycans, can provide sequence and some implied branch structure information (Kawasaki et al., 1999; Jankowska & Cipollo, 2011). Linkage information is limited although can be enhanced significantly using Na^+^ or other metal adducts (Harvey, 2000). Negative ion mode analysis of reducing end derivatives can also be used. In this case CID fragmentation can yield significant cross‐ring ion abundance to allow some linkage and branch assignment (Wheeler & Harvey, 2000; Sagi et al., 2002; Takegawa 2005a, b; Jankowska & Cipollo, 2011). However, unlike permethylated derivatives, there is no mass marker to identify branch points and some fragments derived from cross ring cleavage can have the same mass despite having different identities resulting in some ambiguity. Rudd et al. (2001) have developed approaches utilizing multidimensional chromatographic separation (Doherty et al., 2012). Using sequential glycosidase digestions and glycan chromatographic libraries, accurate structural assignments can be made. Spectral libraries can be used to potentially provide accurate structural matching in native glycans, reducing end derivatives, and other derivatives when coupled with appropriate informatics (Ashline et al., 2014; Campbell et al., 2014; Abrahams, Campbell, & Packer, 2018; Remoroza et al., 2018).

Glycans can also be analyzed in native form after release. These approaches can be rapid and uncomplicated by chemical derivatization procedures and associated purification steps (Hua et al., 2011; Yang et al., 2017). Study of native glycans can be useful in comparative glycomics studies, although the nature of the monosaccharide components can bias signal intensities reported by the mass spectrometer compared with permethylated forms. Although there is no strong UV absorbance moiety or fluorophore, the N‐acetyl bond of monosaccharide components can provide a low level of absorbance allowing for high‐performance liquid chromatography (HPLC) separations if sufficient quantities are analyzed. Native glycans can be separated by high resolution HPLC resins such as porous graphite. However short gradients are used as the reducing end, which will mutarotate between alpha and beta forms, can result in their separation. This situation can be avoided by reduction, which can be performed rapidly and is easily incorporated into purification schema (Chu et al., 2009). Dissociation via CID in positive mode will yield mostly glycosidic cleavages (Jankowska & Cipollo, 2011). Dissociation in negative mode can result in, not only glycosidic but, significant cross ring fragment ion abundances leading to linkage and branch configuration assignments similar to reducing end derivatives (Pfenninger et al., 2002; Reis et al., 2003; Lattova et al., 2005; Zaia et al., 2007). Using hydrogen adducts, abundant fragments are produced retaining both the reducing and non‐reducing ends. This differs from some reducing end derivatives which retain charge on the substituting amine moiety and thus predominantly produce reducing end fragments in positive mode (Jankowska & Cipollo, 2011). Finally, an added advantage over essentially all derivative forms is that the native glycans retain their native configuration and active center reducing end allowing for downstream chemistries if the study requires it.

Released viral glycans have been analyzed by LC/MS with ion mobility separation (IMS) (Hussain et al., 2015). Essentially these methods produce separation of ions dependent on their collision cross‐section and via migration through an electric field opposed by a drift gas. The analytes separate according to their relative mass, size, shape, and charge. An advantage of this method is in its ability to separate glycan ions from impurities making analysis of glycans possible from minimal amounts of material. IMS in negative polarity ionization mode has been used to characterize glycan compositions and their isomers in HIV glycoproteins to reveal overall glycosylation profiles and level of glycan secretory processing in regions of multimerization of HIV gp120 and gp41 (Behrens et al., 2016).

Sialic acid is a key structural component central to many biological functions. Many viruses target SA in specific structural contexts which are often dependent upon the linkage of the SA. A range of chemical labeling strategies have been developed over the past 10 years that allow identification of SA linkage within glycans and glycopeptides (Wheeler, Domann, & Harvey, 2009; de Haan et al., 2015; de Haan, Reiding, & Wuhrer, 2017; Yang, Franc, & Heck, 2017; Yang et al., 2018b; Pongracz, Wuhrer, & de Haan, 2019). These chemical modification strategies function to stabilize the SA, provide a mass tag that can be non‐specific or specific to the SA linkage, and increase ionization potential. Methods are reported that perform the derivatization in both solution and solid phase. These methods have been recently reviewed (de Haan et al., 2020). Essentially, the carboxyl is either esterified or amidated. The functional group added can be chosen to match up or downstream processes to some degree by matching activator and catalyst for the targeted derivative. The p‐toluidine amide, for instance, can be used as a nonspecific SA modification or subsequent to esterification to allow identification and quantitation of Sialylα2,3 or Sialylα2,6 residues in oligosaccharides or glycopeptides (Yang et al., 2018b). Modification of intact glycopeptides can lead to somewhat complex spectra as amino acid carboxyl side chains and termini can also be esterified and amidated. By‐product formation due to the partial loss of water via lactam or lactone formation within the peptide portion may also occur. Identification of these modifications can be programmed into the data search (Yang et al., 2018b).

### B Glycopeptide Analysis

**Figure 4:**
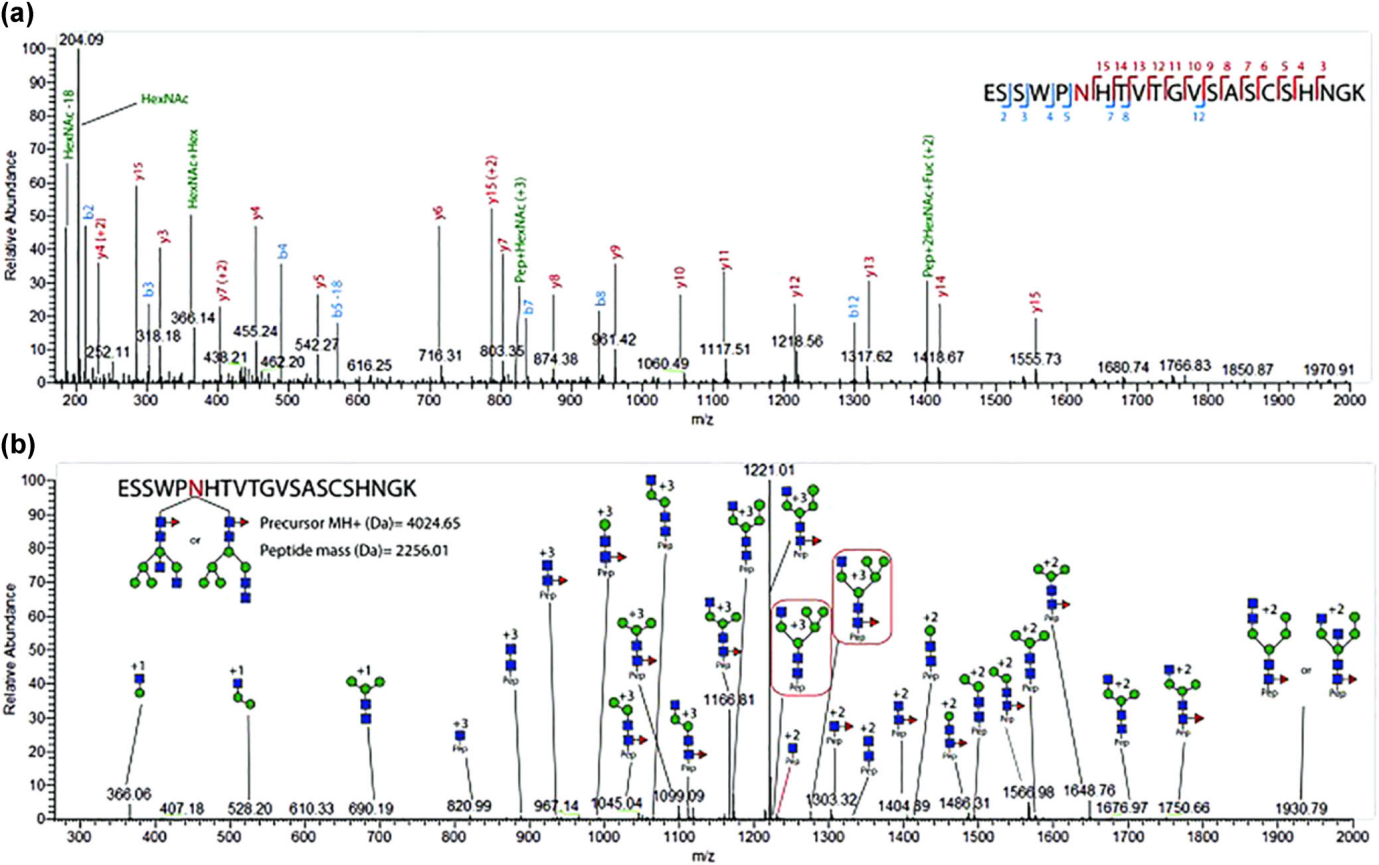
Representative collision‐induced dissociation (CID) and higher energy collisional dissociation (HCD) spectra of an influenza hemagglutinin (HA) glycopeptide (Cruz et al., 2018). (**a**) HCD spectrum of glycopeptide ESSWPNHTVTGVSASCSHNGK. The spectrum contains characteristic diagnostic oxonium ions and the presence of b and y ions derived from the peptide backbone. (**b**) Complementary CID spectrum of the same glycopeptide with a HexNAc_4_Hex_5_Fuc_1_ glycan. Sequential glycan fragmentation is predominant in the spectrum and allows for the glycan composition assignment. Figure reprinted with permission from Cruz et al. (2018). [Color figure can be viewed at wileyonlinelibrary.com]

Figure 3 diagrams a range of pathways used in glycopeptide analysis. These are highlighted in red and green in the figure. Enriched viral glycopeptides have been analyzed by CID, HCD, ETD, MS^E^ as shown in the figure and combinations thereof have been used. The most successful approaches are optimized to yield fragmentation across the peptidyl and glycosyl moieties of the glycopeptide. Because the bond energies of these two moieties differ significantly, with the former being more energetic, CID can be difficult to tune for optimal fragmentation of both. In such cases sequential scans using different collision energies can be applied if possible but this is limited by the available duty cycle time. Collision energy (CE) can be optimized to favor fragment type ion intensities and such strategies can be used, for instance, in MRM approaches to increase method sensitivity (Zhang et al., 2012). Lower CE has been used to generate glycosyl Y‐fragments, retaining glycan compositional information. This was an advantage over higher collision energy where Y ion intensity was lost in favor of oxonium ion intensity, the latter of which suffers from lower specificity and coincident ions. A similar CID optimized CE approach using Q‐ToF MS/MS has been used in the study of influenza B/Malaysia/2506/2004 HA (An & Cipollo, 2011). An Orbitrap HCD/CID fragmentation strategy can be optimized to provide glycopeptide spectra with either increased peptidyl or glycosyl fragment ion abundances leading to improved identification. Example HCD spectra of HIV gp120 glycopeptides is shown in Figure 4A where primarily peptidyl fragmentation has occurred. Alternatively, in Figure 4B, CID is used to generate primarily glycosidic fragmentation (modified from Cruz et al., 2018). Note that combined, this alternating scanning mode approach provides, glycosyl, peptidyl, oxonium, and peptide + core fragments providing a rich assortment of structural identifiers. While HCD and HCD hybrid techniques can be considered an improvement over traditional CID approaches for glycopeptide analysis, other methods are also available that increase information content which may be preferred in some cases.

**Figure 5:**
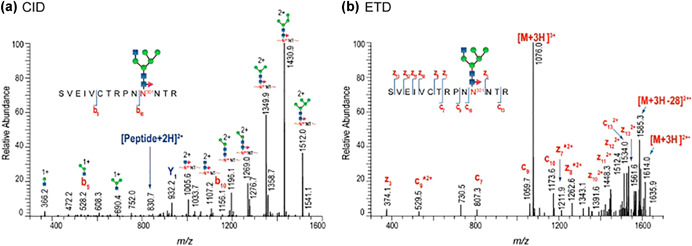
Representative collision‐induced dissociation (CID) and electron transfer dissociation (ETD) spectra of human immunodeficiency virus (HIV) gp140 (C97ZA.012 ‐FT) (Go et al., 2017). (**a**) CID data of glycopeptide SVEIVCTRPNN(Man_5_GlcNAc_2_Fuc_1_)NTR showing the glycosidic cleavages. Y1, oxonium and glycosyl fragments are prominent. Limited peptidyl b and y ions observed. (**b**) ETD data of the same glycopeptide showing the peptide backbone cleavages and deduced glycosylation site located at the second N residue from the N‐terminus. The c and z ions are prominent. Figure reprinted with permission from Go et al. (2017). [Color figure can be viewed at wileyonlinelibrary.com]

As previously stated, ETD can be used to locate the glycosylation site whilst leaving the glycan moiety intact. This is especially useful when viral proteins are highly glycosylated. For example, influenza HA tends to gain N‐glycosylation sites over time as the virus adapts to the human host. In such cases ETD or other strategies, such as multi‐protease approaches, must be employed to locate glycosites and their glycan compositions in peptides with more than one glycosite. O‐glycosylation also tends to occur in stretches and ETD is a preferred method of analysis is cases where such glycopeptides are expected. This process fragments the peptide backbone while sparing most PTMs including glycans. A work‐flow to ETD analysis in viral glycoproteins is shown in Figure 3 outlined in green. ETD and CID can be used in combination to yield site of occupancy information and high quality fragmentation spectra containing peptidyl and glycosyl daughter ion abundances for identification (Saba et al., 2012; Singh et al., 2012). An example of this combination is shown in Figure 5 modified from Go et al. (2017). Figure 5A shows CID spectra where glycosidic bonds are lost resulting in a series of fragment ions with intact peptide allowing for glycan sequence assignment. Figure 5B shows ETD spectra where key peptide fragment ions including c_9_ and z_3_ localize and confirm the position of the glycan substitution.

**Figure 6:**
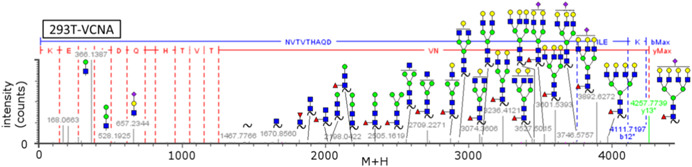
Example MS^E^ spectrum of influenza hemagglutinin (HA) glycopeptide N(NeuAc_1_Gal_4_GlcNA_4_Man_3_Fuc_1_GlcNAc_2_)VTVTHAQDILEK from A/Mallard/Denmark/64650/03 (H5N7) (Parsons et al., 2017). The full glycan attached at each site is shown to the far right. Red lines are y ions and blue lines are b ions. The green line denotes the parent glycopeptide ion. Gray lines are fragments unassigned by BiopharmaLynx. Glycopeptide and oxonium ion assignments made by GLYMPS have been added to the spectra. Oxonium glycosyl, peptidyl, core + HexNAc (Y1) and other core Y ions are prominent. Reprinted with permission from Parsons et al. (2017). Copyright 2017 American Chemical Society. [Color figure can be viewed at wileyonlinelibrary.com]

Another alternative approach is to use MS^E^ (see Fig. 3 outlined in red). As described previously, this technique can be tuned to produce abundant glycosyl and peptidyl fragment ion abundances as seen in Figure 6 taken from Parsons and co‐workers where influenza H5 HA was analyzed (Parsons et al., 2017). However, site of occupancy in peptides with more than one glycosite is difficult to assign and other analyses must be used as previously discussed (Gatlin et al., 2000). MS^E^ and SWATH‐based mass spectrometry allow for data‐independent acquisitions. While no glycoproteomics applications of SWATH in viral glycoprotein analysis has been reported, MS^E^ has been used in the analysis of HIV, influenza, and coronavirus glycoproteins (Xie et al., 2011; Alymova et al., 2016; An et al., 2013, 2015, 2019; Ivleva et al., 2019; Parsons et al., 2017, 2019). Incorporation of all the information provided in MS^E^, that is, abundant peptidyl, glycosyl, oxonium, Y_1_ fragments, into assignment strategies can lead to high confidence assignments.

### C Site Occupancy Analysis

Placement of site occupancy analysis in a generalized work‐flow can be seen in Figure 3 outlined in green. Originally, site occupancy percent was estimated through the use of PNGase F catalyzed N‐glycan release in normal water (Carr & Roberts, 1986). During the enzymatic process asparagine (Asn) is converted to aspartate (Asp) resulting in a ~1 amu increase in mass of the peptide. However, this relatively small change in mass was not easily resolved from complex spectra in early instrumentation. Additionally, spontaneous deamination of Asn to Asp can occur. Thus, even with the more modern high resolution instruments percent occupancy estimates can be misleading, especially in peptide regions rich in Gly where spontaneous deamination can occur at higher probability (Stephenson & Clarke, 1989). To overcome these issues, enzymatic deglycosylation can be performed in ^18^O water so that heavy oxygen in incorporated at the β‐carbonyl group of newly deglycosylated Asn (Asp*). The process was first described by Gonzalez et al. (1992). The converted Asp* will appear 3 amu larger than the cogent unsubstituted Asn form. Those Asp that resulted from spontaneous deamination reside at the +1 position and are therefore identifiable. These methods have been used extensively in viral protein glycoproteomics. In the vaccine context, such analyses can reveal differences in glycan occupancy within predicted antigenic regions as demonstrated with influenza HA grown in different cell systems where HA produced in insect cells had sites more highly occupied than those generated in egg or HEK293 cell lines (An et al., 2013).

Site occupancy analysis of O‐glycosylation sites is highly challenging. A chemical approach is to use β‐elimination with deuterated NaBH_4_. This method was first described by Rademaker and co‐workers in 1993. The process results in the release of O‐glycan alditols as well as generation of peptide backbone with conversion of threonine into dehydroamino‐2‐butyric acid and serine to dehydroalanine, which can be followed by Michael adddition. Mirgorodskaya et al. (2001) developed a β‐elimination procedure via reaction of glycopeptides with NH_2_CH_3_ vapor yielding methylamine derivatives of the Ser and Thr residues with a +13 Da mass tag relative to the unsubstituted counterpart. These chemical means of O‐glycopeptide tagging occupancy studies have not been pursued in the mainstream but remain promising and warrant further investigation.

Recently an O‐glycoprotease became commercially available. The enzyme, OpeRATOR, appears to be broadly active on O‐glycosylation sites occupied by multiple core types. It generates a new N‐terminus by cleavage of the peptide bond between the occupied Ser or Thr and its N‐terminal amino acid residue (Yang et al.,  2018a, c). The enzyme has been used successfully to investigate Zika virus envelope E and NS‐1 proteins (Yang et al., 2018a). Up to an eight‐fold increase in O‐glycosite identifications were revealed in control glycoproteins compared to literature reports using alternative methods. While methods for site occupancy percent estimates have not specifically been devised for O‐glycosylation that leverage this enzyme, the sites of occupancy are better revealed with its use.

## VII VIRUS GLYCOPROTEINS AS A GUIDEPOST FOR GLYCOPROTEOMICS DEVELOPMENT

A wide range of viruses have been studied using glycomics approaches (Harvey, 2018). By far, the most work has been done on two viruses, HIV and influenza. HIV studies have primarily focused on HIV Env proteins gp120 and gp41 and their variants, while influenza studies have focused on HA and to a lesser extent NA. These proteins have inherent properties that have tested glycomics approaches over time. If fact, it is fair to say that the study of viral proteins has aided in the advancement of glycoproteomics methods and some key studies will be described. The studies have been arranged chronologically allowing for both the advancement provided by glycomics and glycoproteomics analysis in virology and in mass spectrometry analysis to be related.

### A Glycomics and Glycoproteomics of HIV spike proteins

**Figure 7:**
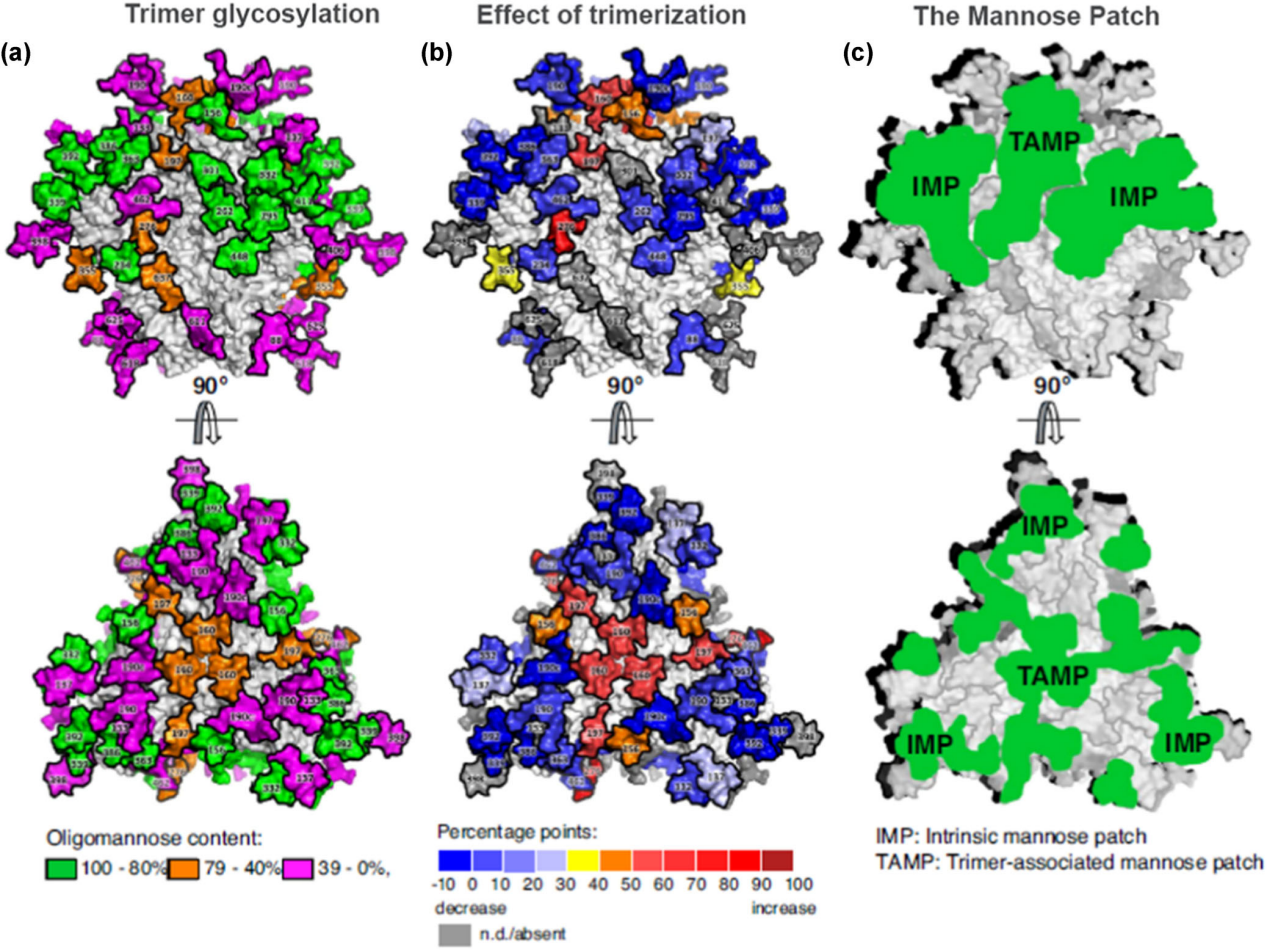
The trimer‐associated mannose patch of human immunodeficiency virus (HIV) gp120 (Behrens & Crispin, 2017). (**a**) The glycans are colored according to their oligomannose content. (**b**) Heat map of the effect of native‐like trimerization on the percentage point of oligomannose‐type glycans of recombinant gp120. (**c**) Schematic of the localization of the intrinsic and the trimer‐associated mannose patches on Env. Figure reprinted with permission from Behrens & Crispin (2017). [Color figure can be viewed at wileyonlinelibrary.com]

The envelope spike (env) protein is essential for HIV attachment to host cell surface receptors and is composed of gp120 and gp41. It is displayed on the virion surface as a trimer of heterodimers covered with a dense carbohydrate coat which accounts for about half of its mass. The gp120 protein of ~480 amino acids can have up to 27 N‐glycosylation sites and many of them are densely organized, providing for difficult analytical challenges. The protein also has a single O‐glycosylation site which may be required for host cell invasion (Hansen et al., 1996). Over the past two decades some key glycosylation features of the env protein have been revealed. The protein is highly mutable and tends to gain glycosylation sites on epitopes recognized by broadly neutralizing antibodies (bNAbs) (See Fig. 7 and Behrens & Crispin, 2017). The trimer has three regions dense in high mannose glycans: two in regions intrinsic to monomers and one in trimer associated regions. The protein has been studied in soluble native‐like trimers (Behrens et al., 2016; Guttman et al., 2014), membrane‐associated trimers (Go et al., 2015), and virion‐derived (Panico et al., 2016) forms. Glycosylation differences have been noted across the forms. Env is recognized by a number of host cell lectins such as dendritic mannose receptor (Gringhuis et al., 2010) and DC‐SIGN (Gringhuis et al., 2010) which then facilitate infection. Below are some key glycomics studies that aided in the understanding of HIV env glycosylation and its key features that may govern immune system recognition and rational vaccine design.

Glycomics analysis of the env protein appeared in 1990, shortly after its discovery (Sattentau & Weiss, 1988; Syu et al., 1990). Leonard et al. (1990) investigated Chinese hamster ovary cell expressed gp120 of HIV‐1 using an FAB‐MS approach. The strategy involved proteolytically processing glycopeptides and sequential treatment strategies with endo‐beta‐N‐acetylglucosaminidase H (endo H) and peptide: N‐glycosidase F (PNGase F). Endo H releases only high mannose and hybrid N‐glycans whilst PNGase F releases most known mammalian N‐glycans. The differential release strategy allowed glycan subclasses at specific glycosylation sites to be determined. All 24 N‐glycosylation sites were found to be occupied. 11 sites were occupied primarily by high mannose glycans and 13 by complex type glycans. This study also described the complex disulfide bond network in gp120 using a multi‐protease strategy coupled with PNGase F release and reducing agents. Glycopeptide analysis was performed by both Edman degradation and FAB‐MS. While detailed compositions and heterogeneities of individual glycosites were not reported, this study is a landmark as it first defined what would become the high mannose portion of the glycan shield. This region interacts with host receptors such as DC‐SIGN on dendritic cells through the receptor's recognition of the high mannose glycan patch. Expanding on their work, Geyer and co‐worker investigated gp120 of HIV‐2 released glycans using MALDI‐ToF MS analysis revealing that the abundances of glycan sub‐types vary with cell type used to generate the protein (Liedtke, Geyer, & Geyer, 1997).

Cutalso and co‐workers studied HIV‐I (SF2) gp120 using nanoLC/MS Q‐ToF analysis. Identifications were primarily made based on parent ion mass matching coupled with CID fragmentation analysis (Cutalo, Deterding, & Tomer, 2004). Very few peptide bond fragmentations were observed making assignment primarily based on mass match to ion abundances that were consistent with glycosidic cleavage with intact peptide. Twenty‐five of 26 N‐glycosites were identified, 13 of which were occupied by high mannose and 12 by complex glycans. Eight sites contained sialylated glycans. Two sites previously reported to be primarily high mannose were found to contain complex glycans.

Irungu et al. (2008) compared HPLC/ESI‐FTICR MS and MALDI‐ToF/ToF MS in the analysis of HIV‐1 gp140DeltaCF1 envelope protein, which has 31 potential N‐glycosylation sites. The two approaches were complimentary. Over 130 unique glycan compositions were identified by each technique, 90 of which overlapped, for a total of 350 glycopeptide compositions. The LTQ FTICR approach produced few peptidyl fragments but did generate Y1 fragments from most glycopeptides. This fragment contains core HexNAc + peptide backbone and is usually abundant and diagnostic. MALDI‐ToF/ToF, on the contrary, produced information dense spectra rich in fragment ion abundances generated from peptide cleavages but had little glycosidic fragment ion abundance and therefore little glycan sequence data. In efforts to better cover glycosylation heterogeneity the same group analyzed the same gp140DeltaCF1 using positive and negative polarity modes in MALDI‐ToF/ToF subsequent to three different enrichment protocols including reverse phase HPLC, Sepharose SEC, and lectin enrichment (Zhang, Go, & Desaire, 2008). Unsurprisingly, cumulative data from both polarity modes gave the best results in terms of detected glycopeptides and glycoforms. This was especially the case when mixtures of glycopeptides included both neutral and acidic glycoforms. Assignments were facilitated by an in‐house developed glycomics software, GlycoPep DB (Go et al., 2007).

Go et al. (2008) investigated two HIV‐1 env protein constructs, JR‐FL gp140ΔCF and CON‐S gp140ΔCFI, referred to from here forward as JR‐FL and CON‐S. MALDI ToF/ToF and LC/ESI‐FTICR MS were used in the study. This study links structural aspects of the envelope proteins to immune responses emphasizing glycosylation. Glycopeptide and de‐glycopeptide (site occupancy) studies were carried out. The proteins had 27 and 31 N‐glycosylation sites respectively. CON‐S elicited neutralizing antibody response spanning three M clades of the virus when used in DNA/recombinant protein vaccine in animal models while JR‐FL was described as a poor antigen (Liao et al., 2006). The more immunogenic CON‐S had more unutilized glycosylation sites specifically surrounding the V1, V2, C2, and V3 regions as well as smaller and less processed glycans in C2, V3, and V4. CON‐S also had more high mannose glycans near the N‐terminus of the protein, possibly providing more rigidity and protein stability. The V1/V2, and V3 loops flank the receptor binding site, a prime region for antibody recognition. Using the combined instrument approach, the authors report over 300 glycopeptide compositions per antigen. A high degree of detected glycosylation heterogeneity, site occupancy analysis, and use of in‐house developed GlycopepID software provided for characteristics of these antigens to be revealed. Site occupancy, glycan sub‐type, and placement correlated with differences in antigen fitness although differences in amino acid sequence, such as those in the V1–V5 region of the proteins, cannot be ruled out as contributing factors. Additionally, the proteins were enriched using lectin affinity and such approaches can introduce glycoform bias (Nilsson et al., 2013). The authors extended their research to a comparison of the related C.CON and primary isolate C.97ZA012. These proteins were immunogenically similar. Comparisons supported the notion that high mannose glycosylation in the V3 and surrounding regions coupled with unoccupied glycosylation sites correlated with good Clade C immunogenicity (Go et al., 2009).

Glycosylation patterns of transmitted/founder versus chronic infection HIV‐1 envelopes differ (Go et al., 2013). An enzymatic approach that includes an up‐front complete or partial enzymatic deglycosylation step before trypsin digestion has been used to compare representative founder/transmitted envelope glycosylation patterns similar to the approach used by Leonard et al. (1990) discussed earlier. The approach was reported to maximize the N‐glycosylation coverage. Initial attempts using the more conventional approach detected only 40% of glycosylation sites and 30% of glycan compositions compared to the multi‐enzyme simplification approach. Using FTICR mass spectrometry the authors reported complete coverage of glycosites. Compared to recombinant env proteins from viruses derived from chronic HIV‐1 infections, those from transmitted/founder proteins (both clades B and C) displayed marked differences in their glycosylation site occupancies and in amounts of complex glycans. This is a critical difference since cell receptor recognition requires high mannose glycan in the variable region, specifically for DC‐SIGN and DC‐SIGNR binding (Lin et al., 2003). A drawback of the study was that the fine structure of glycans was not revealed due to the enzymatic simplification. However, the biomedical question revolved around site occupancy and site‐specific distributions of high mannose/hybrid versus complex glycans, all of which were addressed by the approach. The same group compared transmitted/founder env gp120 produced in CHO and HEK293 cells. Using a combination of CID and ETD fragmentation with LTQ FT and LTQ Orbitrap, this study focused on both N‐ and O‐glycosylation. HEK293 cells produced core‐1, ‐2, and ‐4 while CHO produced only Core‐1 O‐glycans. Complex N‐glycans were higher in abundance in HEK293 and CHO cells produced phosphomannan at site N197 in the V2‐C2 region.

**Figure 8:**
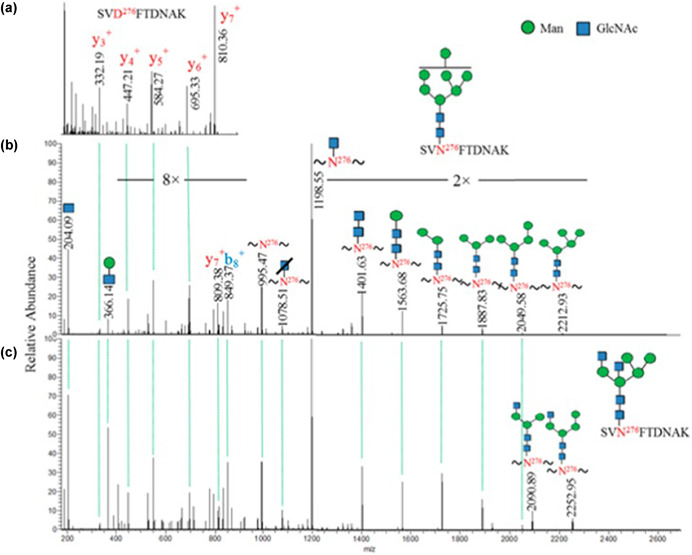
Representative MS/MS spectra for identification of N‐linked glycopeptides using the higher energy collisional dissociation (HCD) spectral‐aligning strategy described by Yang et al. (2014). (**a**) MS/MS spectrum of deglycosylated peptide SVD276FTDNAK is used to provide experimentally identified b‐ and y‐ions. (**b**) MS/MS spectrum of a glycopeptide with matched pattern of b‐ and y‐ions to that of deglycosylated peptide. Additional b‐, y‐, and Y‐ions facilitate identification of the glycopeptide SVN276FTDNAK with Man_7_GlcNAc_2_, which was used as a spectral template to identify glycopeptides with the same peptide backbone but different glycan Man_5_GlcNAc_4_ shown in (**c**). Figure reprinted with permission from Yang et al. (2014). [Color figure can be viewed at wileyonlinelibrary.com]

A spectral aligning strategy has been developed for use in HIV gp120 glycoproteomics analysis (Yang et al., 2014). The method leverages Orbitrap HCD analysis fast duty cycle and high energy fragmentation patterns. The strategy was as follows: (1) deglycosylated peptides and glycosylation sites were identified from PNGaseF treated samples; (2) b‐ and y‐ions of peptide backbones were identified using the deglycosylated peptide spectra; (3) MS/MS spectra containing oxonium ions were extracted from the raw file of the sample without PNGase F treatment; (4) the b‐ and y‐ions of the deglycosylated peptides were used to filter the MS/MS spectra of glycopeptides; (5) peptide and Y‐ions (Peptide + HexNac) of glycopeptides MS/MS spectra were used to further filter putative identifications; (6) the best‐matched MS/MS spectra of glycopeptides were used to cross‐match the other MS/MS spectra of glycopeptides with the same peptide backbones; (7) finally, the precursor *m*/*z* and the delta mass between the peptide moieties and glycopeptides were used to determine the glycoforms. Samples of approximately one microgram were analyzed. An example of the coalignment identification of two different glycopeptides with the same peptidyl but different glycosyl moieties is shown in Figure 8. The concept relied heavily upon the presence of peptide b‐ and y‐ions in the deglycosylated and non‐treated samples, which are then used in a filtering approach of spectra containing oxonium ion in tentative glycopeptide spectra. The resultant pool of glycopeptides candidate spectra are then tempered by further cross reference to Y‐ion presence and the glycan moiety is predicted via the difference between the predicted peptide mass and the delta mass. Among the 24 predicted N‐glycosylation sites 19 were identified. Reported were 2119 manually verified glycopeptide spectra. The single O‐glycosylation site was also identified. While PNGase F deglycosylation was performed, which should allow for site occupancy estimates, no site occupancy estimates were reported. Therefore, it is not known to what extent the predicted sites were occupied. The approach was performed on HIV‐1 latently infected T‐cell line, ACH‐2 cell derived gp120. The ACH‐2 cell derived samples may potentially have glycosylation patterns seen in natural infections, although this is not confirmed. With limited sample amounts, five N‐glycosylation sites were identified as well as the O‐glycosylation site. While glycosylation site and heterogeneity detection may have been less than optimal this novel approach is promising. Further optimization, for instance, using enrichment techniques, multiple polarity mode analysis, multiple instrument platforms, glycosidase reduction of complexity, and/or further optimization of b‐ and y‐ion formation could harness the power of this strategy.

Behrens and co‐workers analyzed a soluble trimeric form of HIV‐1 env BG505 SOSIP.664 composed of three gp120 proteins. The trimeric form is difficult to isolate which may explain why most functional studies, that is, showing antibodies binding the high mannose glycan patch, are conducted with the monomeric form (Calarese, 2003; Walker et al., 2011). Glycosylation site oblation and a panel of monoclonal antibodies (bNAb) were used to interrogate the role of individual glycosylation sites on antibody recognition and influence on neighboring and distanct glycosylation site compositions. The study combined MALDI ToF/ToF and LC/MS Orbitrap with HCD for glycopeptides and Q‐ToF IMS and HILIC HPLC for released glycan analysis. A glycan library was constructed from identified glycans for use in glycopeptides analysis. Site specific quantitation was performed by summing of ion intensities. Proteolytic digestions used both trypsin and chymotrypsin. The data reported revealed no glycopeptides with more than one glycosylation site making position assignment less ambiguous than might be expected on average for such densely glycosylated proteins. Both gp120 and gp41 were analyzed. Intriguingly the MALDI ToF and LC/MS methods gave highly comparable estimates of glycan compositions at specific glycosylation sites. A model of the fully glycosylated trimer based on the PDB‐5ACO structure was constructed providing a map of glycosylation based on the processing states detected. Complex glycans were reported at the C‐ and N‐termini. Glycans in interprotomer regions were reported to exhibit limited processing. Glycans at the trimer base tended to be complex. A model of regionally specific glyco‐types was constructed (See Fig. 7). Glycosylation site knockouts of most sites in the glycan patch were studied for changes in monoclonal antibody neutralization. In general, when glycans known to be key components of epitopes were absent, bNAb sensitivity was substantially reduced or lost entirely. In terms of glycosylation and glycosite knockouts, the N332A mutant (glycosite knockout) was reported to redirect glycosylation at neighboring sites of the glycan patch but only subtly from Man_9_GlcNAc_2_ to Man_8_GlcNAc_2_. Knockout of sites N332 and N137 had profound effects on mannan processing leading to smaller high mannose glycans. There were many implications for bNAb specificities regarding glycan subtype, overall structures, and interaction between glycosylation sites.

Go et al. (2017) investigated eleven HIV‐1 Env proteins in a tour de force glycomics effort employing Orbitrap mass spectrometry with CID and ETD analysis in alternating scans and data dependent acquisition. Data were processed using GlycoPep DB (Go et al., 2007), GlycoPep ID (Irungu et al., 2007), and GlycoMod (Cooper, Gasteiger, & Packer, 2001). Overall the data were of high quality and apparently dense coverage of glycosylation sites across all proteins. Glycopeptides with more than one occupied glycosylation site were reported as their summed glycan composition. It was not clear how effective ETD experiments were to discern multiple glycoforms in these cases. In addition to the proteins analyzed in this study, the data were also compared to that of several other gp120 and gp140 proteins available in the literature (Go et al., 2015; Behrens et al., 2016; Panico et al., 2016). The gp120 protomers analyzed were all trimeric forms. The env proteins investigated differed in construct design, the purification method, and the producer cell type. Both membrane bound and soluble forms were studied. Remarkably, the glycosylation patterns of env trimers were similar even though the genotypes, construct designs, producer cells, and purification methods differed. The glycan patch was unanimously high mannose (sites N156, N262, N334, N389 to N448, and N463) in all the trimers. Likewise, some sites were consistently occupied with highly processed glycoforms (N187, N197, N230 to N234, N356, and N386). These two glycosylation regions were described as the “consensus” profile. Glycosylation sites outside of these eleven consensus sites differed considerably. Overall this study may be informative for vaccine developers since the “consensus” regions were identified in the trimeric env forms. Glycans are targeted by the humoral response and these patterns were made clearer in this study. Cell producer differences in glycosylation site glycan subtype were identified. Interestingly, differences in clustering of glycosylation sites (N230, N234, and N242) impacted glycan subtype in some regions.

### B Glycomics and Glycoproteomics of Influenza HA

**Figure 9:**
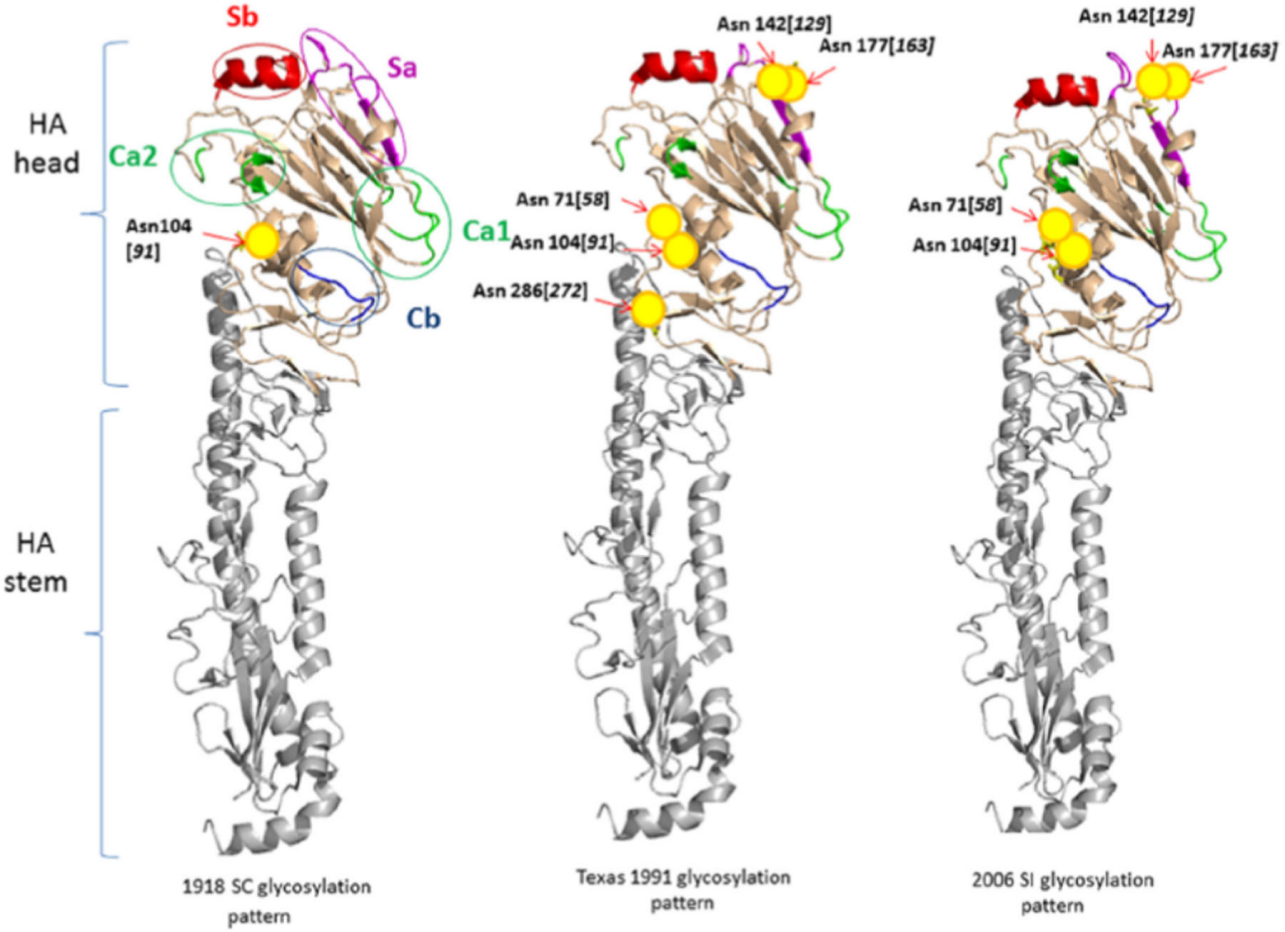
The glycosylation patterns of selective strains of human H1N1 viruses (Sun et al., 2013). Ribbon diagrams of monomeric H1 hemagglutinin (HA) structures were illustrated using 1918 SCHA0 as the template (PDB entry, 1RD8). The italicized numbers inside brackets are based on an H1 HA crystal structure (1RUZ) numbering. The antigenic sites Sa, Sb, Ca, and Cb are colored in magenta, red, green, and blue, respectively. The potential glycosylation sites on the HA of 1918 SC, Texas/91, and SI/06 virus are shown in yellow circles, with the corresponding Asn labeled. The gain of glycosylation sites in antigenic regions of the HA head are exemplified. Figure reprinted with permission from Sun et al. (2013). [Color figure can be viewed at wileyonlinelibrary.com]

Influenza A (IAV) virus is divided into subtypes based on the HA and NA proteins. There are 18 HAs and 11 NA proteins. Influenza B virus (IBV) also infects humans and is broken down by lineage and strain but not divided into subtypes (Zambon, 1999). Seasonal IAV strains that infect humans are primarily H1N1 and H3N2. The seasonal vaccine usually contains one strain each of these and one or two strains of IBV. Pandemic strains typically arise from either swine or avian species. Glycosylation patterns across subtypes differ and change through genetic shift and genetic drift as they arise in and propagate through the human populations. HA exists as a trimer and higher order multimers. It is divided into a stem and a globular head region (Fig. 9). The head region of HA tends to gain glycosylation sites that co‐localize with antigenic sites (Arinaminpathy & Grenfell, 2010; Sun et al., 2011). Figure 9 demonstrates how glycosites were added in antigenic sites Sa and Ca1 over time. Influenza is bound by host cell collectins such as SP‐D in the lung, DC‐MR, and DC‐SIGN (Veldhuizen, van Eijk, & Haagsman, 2011). These interactions play a role in a complex network resulting in immune system modulation, infection, and pathogen removal. SP‐D, DC‐MR, and DC‐SIGN all recognize high mannose glycans while others such as galectins and ficolins recognize other glycan types. Glycomics characterization at the glycosylation site level is informative of which innate immune molecules might interact with different HA subtypes during an infection (York, Stevens, & Alymova, 2019). Conversely, influenza HA recognizes host cell carbohydrates that terminate with SA. While viruses that primarily infect humans recognize Sialylα2,6‐linked SA, avian viruses typically recognize Sialylα2,3, and swine viruses recognize both Sialylα2,3 and Sialylα2,6. Therefore, assay of HA binding preferences can be informative and this will be discussed later in Section V. Below are some key glycomics studies of influenza presented in chronological order to emphasize technologies used, their advancement and biological implications of the information revealed. A list of glycomics studies in influenza up to 2017 can be found here (Harvey, 2018). Additional, more recent work can be found in Table 1.

**Table 1:** Mass spectrometric methods used for analysis of glycoproteins from viruses from 2017 through 2019

Glycoprotein	Expression system	Preparation	MS Methods	MS outcome	Identified glycans		Glycosylation sites	Reference
					N	O		
Adeno‐associated virus capsid	HEK293	Trypsin, GluC, PNGaseF	MALDI‐TOF‐TOF, LC‐MS/MS	SSG, SO, GG	M, H, C	na	N499	Aloor et al. (2018)
Ebola GP from 3 variants	HEK293T	PNGaseF, fluroescent label	MALDI‐TOF/TOF	GG	M, H, C	na	na	Fujihira et al. (2018)
Ebola GP_1,2_ from 5 variants	HEK293T	PNGaseF, beta‐elimination, permethylation	MALDI‐TOF	GG	M, H, C	10 different types	na	Collar et al. (2017)
Flu candidate vaccines HA, NA	Egg	Trypsin, AspN, chymotrypsin, proteinase K, pepsin	LC‐MS/MS	SSG	M, H, C	na	HA:N28, 40, 104, 136, 293, 304, 490, 498;	She, Farnsworth, Li, and Cyr (2017)
							NA:N50, 58, 68, 146, 235, 386	
Flu H1N1 HA	Egg	Trypsin	LC‐MS/MS	SSG	M, H, C	na	N28, 40, 71, 104, 142, 177, 304, 498	Cruz et al. (2018)
Flu H1N1 HA	Egg	2D gel, PNGase, trypsin	MALDI‐TOF, LC‐MS/MS	Peptide identification	na	na	na	Wu et al. (2018)
Flu H1N1 HA	Baculovirus	Trypsin, AspN, pepsin, PNGaseF	LC‐MS/MS	SSG	M, H	na	N28, 40, 104, 293, 498	Liu et al. (2017)
Flu H1N1 NA	HEK293T, insect Hi‐5	Trypsin, PNGaseF	LC‐MS/MS	SSG	M, H, C	na	N88, 146, 235	Zhu et al. (2019)
Flu H1N1, H3N2, type B HA	Egg, MDCK, Sf9	Trypsin, PNGaseF, permethylation	MALDI‐TOF, LC‐MS/MS	SSG, GG	M, H, C	na	H1N1:N11, 23, 87, 276, 287, 481; H3N2:N8, 38, 45, 122, 133, 144, 165, 246, 285, 483; type B:N24, 58, 144, 166, 302, 331, 490	An et al. (2019)
Flu H3N2 HA1	Egg, MDCK‐SIAT1	PNGaseF, trypsin, elastin	LC‐MS/MS	Identify glycosylation sites	na	na	N8, 22, 38, 45, 63, 126, 133, 158, 165, 246, 285	Beer et al. (2018)
Flu H5N1 HA	SuperMan5, KM 71	endoH	LC‐MS/MS	SO	man5, M	na	N22, 165, 286, 478	Macioła et al. (2017)
Flu H5N7 HA	HEK293T, HEK293S GnT1‐), S2	Trypsin, PNGaseF	MALDI‐TOF, LC‐MS/MS	SSG	M, H, C	na	N11, 23, 165, 286, 480	Parsons et al. (2017)
Flu H9N2 HA, NA	Egg, MRC‐5	PNGaseF	MALDI‐TOF/TOF	GG	M, H, C	na	na	Chen et al. (2017)
Hepatitis C	Huh7.5	Trypsin, PNGaseF	MALDI‐TOF/TOF	GG	H, H	na	na	Guo et al. (2018)
Hepatitis C E2	HEK293, sf9	Trypsin, chymotrypsin	LC‐MS/MS	SSG	M, H, C	na	N417, 423, 430, 448, 476, 532, 540, 556, 576, 623, 645	Urbanowicz et al. (2019)
HIV Env	CHO	Trypsin, chymotrypsin, lysC, PNGaseF, endoH, alpha 1‐2,3 mannosidase	LC‐MS/MS	SSG, SO	M, C	na	N88, 133, 137, 156, 160, 182, 185, 197, 234, 262, 276, 295, 301, 332, 339, 355, 363, 386, 392, 448, 460, 611, 618, 625, 637	Ivleva et al. (2019)
HIV Env	293 F	Trypsin, chymotrypsin, ArgC, elastase, subtilisin, EndoH, PNGaseF	LC‐MS/MS	SSG, SO	M, C	na	N88,133, 137,156, 160,185, 197, 234, 262, 276, 295, 301, 332, 339, 355, 363,3 86, 392, 398, 406, 411, 448, 462, 611, 625, 637	Cao et al. (2018b)
HIV Env	HEK293F	EndoH, PNGaseF, trypsin, chymotrypsin	LC‐MS/MS	SSG, SO, GG	M, C	na	N88, 133, 137, 156, 160, 188, 197, 234, 262, 276, 295, 301, 332, 339, 355, 363, 386, 392, 398, 405, 411, 448, 462, 611, 618, 625, 637	Sarkar et al. (2018)
HIV Env	CHO	PNGaseF, EndoH, trypsin, chymotrypsin		SSG, GG	M, C	na	N88, 156, 160, 188/190, 197, 234, 262, 276, 295, 332, 339, 355, 363, 386, 392, 406, 448, 462, 611, 618, 637	Behrens et al. (2018)
HIV Env	CHO	EndoH, trypsin, chymotrypsin	LC‐MS/MS	SSG, SO	M, C	na	N156,184‐187, 197, 230, 262, 289, 295, 332/334, 356, 386‐396, 397‐412, 442, 448	Go et al. (2017)
HIV Env	HEK293F	PNGaseF, EndoH, trypsin, chymotrypsin, gluC	LC‐MS/MS	SSG, GG	M, C	na	N88, 137, 156, 160, 190, 197, 234, 262, 276, 295, 301, 332, 339, 355, 363, 386, 392, 406, 411, 448, 462, 611, 618, 625, 637	Behrens et al. (2017)
HIV gp120	HEK293F	EndoH, sialidase, PNGaseF, trypsin, proteases	LC‐MS/MS	SSG, SO	M, H, C	na	N88, 130, 132, 160, 188, 234, 241, 262, 276, 332, 339, 356, 413, 448, 463	Hargett et al. (2019)
HIV gp120	MGAT1‐CHO, CHO‐S	Trypsin, PNGaseF, permethylation	MALDI‐TOF	GG	M, C	na	na	Byrne et al. (2018)
HIV gp120	A66‐R5 Tcells, CHO, HEK293F	Trypsin, PNGaseF, endoH	LC‐MS/MS	SSG, SO	M, C	na	N88, 133, 137, 156, 160, 190, 197, 234, 262, 276, 295, 301, 332, 339, 355, 363, 386, 392, 398, 406, 411, 448, 462	Struwe et al. (2018)
HIV gp120	HEK293F	Trypsin, ArgC, elastase, subtilisin, chymotrypsin, EndoH, PNGaseF	LC‐MS/MS	SSG, SO	M, C	na	N88, 133, 137, 156, 160, 185, 197, 234, 262, 276, 295, 301, 332, 339, 355, 363, 386, 392, 398, 406, 411, 448, 462, 611, 625, 637	Cao et al. (2017)
HIV gp120	HEK293F + KIF	PNGaseF, B‐elimination, intact MS	LC‐MS/MS	SO	M	core‐1	N332	Struwe et al. (2017)
HIV gp120	HEK293	Proteases, PNGaseF	LC‐MS/MS	SO	na	na	many	Yu et al. (2018)
Human Cytomegalovirus gB	CHO, BY‐2	Trypsin, chymotrypsin, GluC, PNGase F or A, permethylation	LC‐MS/MS, MALDI‐TOF	SSG, SO, GG	M, H, C	na	N68, 73, 85, 208, 281, 286, 302, 341, 383, 405‐409, 417, 452, 464‐465, 554, 585	Smargiasso et al. (2019)
IBV Beaudette spike	Vero	Trypsin, chymotrypsin, PNGaseF	LC‐MS/MS	SO	na	na	N212, 237, 247, 276, 513, 591, 1051, 1074	Zheng et al. (2018)
IBV M41 spike RBD	HEK293T	Trypsin, chymotrypsin, PNGaseF	LC‐MS/MS,MALDI‐TOF	SSG, SO, GG	M, H, C	na	N33, 59, 85, 126, 145, 160, 194, 229, 246	Parsons et al. (2019)
Lassa GPC	MDCK‐II	PNGase F, trypsin, chymotrypsin, GluC	IM‐ESI, LC/MS/MS	SSG, GG	M, H, C	na	N79, 89, 99, 109, 119, 167, 224, 365, 373, 390, 395	Watanabe et al. (2018)
Newcastle disease HN	Egg	Trypsin, PNGaseF	LC‐MS/MS	SSG	M, H, C	core‐1	N341, 433, 481, T71	Pegg et al. (2017; Qian et al. (2018)
RSV fusion protein	Expi293	Trypsin, GluC, PNGaseF	LC‐MS/MS	SSG	C	na	N70	Qian et al. (2018)
Varicella zoster gE	CHO‐K1	trypsin, pronase, PNGaseF, O‐glycosidase	LC‐MS/MS	SSG	C	core‐1	N437, S79, T329, T333, T512, T519, T526	Nordén et al. (2019)

BY‐2, tobacco bright yellow‐2; C, complex; CHO, Chinese Hamster Ovary; E,Env, Envelope; GG, global glycosylation; GP,gp,g, glycoprotein; GPC, glycoprotein complex; H, hybrid; HA, hemagglutinin; HEK, human embryonic kidney; HIV, human immunodeficiency virus; HN, hemagglutinin‐neuraminidase; Huh, human hepatocellular carcinoma; IBV, infectious bronchitis virus; KM71, *P. pastoris* yeast cells; M, high mannose; MDCK, Madin–Darby Canine Kidney; MGAT1, mannosyl(alpha‐1,3‐)‐glycoprotein beta‐1,2‐N‐acetylglucosaminyltransferase; MRC‐5, medical research council cell strain 5; na, not applicable; RBD, receptor‐binding domain; RSV, respiratory syncytial virus; SF, Spodoptera frugiperda; SIAT1, alpha‐2,6‐sialytransferase; SSG, site‐specific glycosylation; SO, site occupancy; Superman5, human glycoengineered *P. pastoris* cells; Vero, African Green Monkey Kidney cell line.

Mir‐Shekari et al. (1997) were among the first to characterize IAV HA glycosylation sites using mass spectrometry based methods in 1997. They studied the A/WSN/33 (H1N1) virus using a magnetic sector mass spectrometer fitted with a MALDI source. The study used then‐conventional techniques for isolation and identification of glycopeptides and their resident glycans. HA was isolated using an antibody affinity column. Proteolyzed HA peptide fractions were collected by size exclusion chromatography and further separated and isolated by reverse phase HPLC. Glycopeptide compositions were identified by parent mass matching. Glycopeptide specific glycans were released and resident glycans identified. Lectin affinity and exoglycosidase digestions were also used in characterizations. This paper established compositions found in the stalk, intergenic (region between head and stalk) and globular head regions of H1N1 HA. There were six predicted glycosylation sites, four of which were found to contain glycans. Glycans tended to be the largest, most complex forms with tetraantennary branching in the stem closest to the membrane. Glycans in the intergenic region also tended to be complex but were smaller with mostly biantennary branching. The head region had two glycosylation sites, one having mostly biantennary glycans and the other predominantly high mannose. This has been the general regiospecific pattern of glycosylation in H1 HA hence. Site 65, or those close by, which is near the bottom of the head region, has been identified as high mannose a number of times, which is apparently important in host collectin recognition such as with SP‐D (Hartshorn et al., 2008; Qi et al., 2011; Tate, Brooks, & Reading, 2011).

A number of different cell culture systems are used in influenza or influenza protein production. These include mammalian cells such as MDCK (Krammer et al., 2010), Vero (Gränicher et al., 2019), hen egg (Ghyka, Samuel, & Portocala, 1978), insect such as *Spodoptera frugiperda* (sf9) (Rota et al., 1990) and *Trichoplusia ni* (High‐Five) (Krammer et al., 2010), and plant such as *Nicotiana benthamiana* (Maharjan & Choe, 2019) with hen egg as the traditional cell substrate. Each of these cell systems will produce glycosylation compositions and patterns according to their origin's biological niche. Several groups have examined HA or NA glycosylation patterns in these alternative substrates either in stand alone or in comparative studies (Xie et al., 2011; Zhang et al., 2012; An et al., 2013, 2019; Lin et al., 2013; Rödig et al., 2013; Parsons et al., 2017).

Zheng and co‐workers analyzed pandemic strain HA from A/California/04/09 (H1N1) recombinantly produced in *Nicotiana benthamiana* and *Spodoptera frugiperda*. Data were collected on a triple quadupole linear trap using product ion scanning of oxonium ions to trigger fragmentation of glycopeptides ions. Quantitative analysis was performed by MRM for glycoform distributions targeting high mannose forms since they were common across insect and plant samples. Glycosylation sites were verified by analysis of deglycosylated samples by Orbitrap analysis using HCD. Site occupancy estimates were performed without the use of stable isotopes. Four of the five sites were reported to be occupied at an estimated ~95% whilst one site, N11 at the foot of the stalk was estimated at 60%. Both insect and plant cell derived HA occupancies were highly similar. The most striking difference was the abundance of Man_9_GlcNAc_2_, which was ∼60‐fold higher in the plant HAs, while the other glycoforms (Man_5–8_GlcNAc_2_) displayed a ∼three‐fold difference between insect and plant HAs. Paradoxically, the authors reported nearly 20‐fold more apo core peptide (no glycosylation) was found in the plant HAs than in HA1 from insect, suggesting that the glycosylation efficiency was higher in the insect system. However, this seems counter to site occupancy estimates, which were essentially the same for both cell substrates. Strikingly, the plant derived HA had no complex glycans detected. This was likely due to the design of the recombinant protein which had a KDEL ER retention signal peptide that prevented Golgi processing. This approach was proposed as a vaccine strategy to prevent plant like glycosylation patterns.

An et al. (2013) compared HA H5N1 avian viruses produced in egg, High‐Five, sf9, and HEK293 cell systems. One of the sf9 cell lines used was modified to produce humanized biantennary N‐glycans. LC/MS^E^ was used to analyze glycopeptides and released glycans were analyzed by quantitative permethylation analysis by MALDI ToF MS. Permethylation profiles revealed that 20% of HA glycans produced in High‐Five cells contained core Fucα1,3 substitutions, a modification that is associated with allergy in insect glycoproteins (Altmann, 2007; Palmberger et al., 2014). No such modification was seen in sf9 derived HA. Interestingly, all insect cells were glycosylated at two glycosylation sites predicted to be poor glycosites, one of which contained proline at the X position of the NX(S/T) sequon. Neither egg nor HEK293 cell derived HA forms were glycosylated at either of these sites to a detectable level. Both glycosylation sites were in predicted antigenic sites. Therefore, cell substrates may impact vaccine function since glycosylation may not only differ in composition but also occupancy in key antigenic regions of HA. HEK293 cell derived HA had larger complex glycans and, since it was expressed as a recombinant protein and no viral NA was present, the HA also contained SA, which does not occur in nature due to influenza NA activity.

The 1968 pandemic flu strain A/Hong Kong/1/68 H3N2 caused significant morbidity and mortality (Smith et al., 2009). As the virus evolved from season to season in the human populations it gained glycosylation sites, particularly in the globular head region. As influenza evolves and gains glycosylation sites, cytotoxicity can be reduced (Reichert et al., 2010; Wanzeck, Boyd, & McCullers, 2011). An et al. (2015) investigated HA from viruses constructed to represent key glycosylation states of H3N2 strains seen between 1968 and 2002. Four strains of Hong Kong/1/68 H3N2 HA were engineered: wild type with the original seven glycosylation sites and three strains with +1, +2, and +4 historically accrued sites. LC/MS^E^ was used to analyze glycopeptides and released, permethylated glycans were semi‐quantitatively measured by MALDI ToF MS. Site occupancy analysis was also performed. Most sites were predicted to be fully glycosylated. Eighty‐two different site‐specific glycan compositions were observed on the head region. Head glycosylation sites N133, N246, and N165 were all found to contain high mannose glycans exclusively. The N133 and N246 were sites added and their appearance correlated with diminished disease severity in a murine model. The decrease was shown to be associated with lung surfactant SP‐D activity, a collectin that recognizes high mannose glycans and facilitates virus removal from the lung. All head glycosylation sites were found to be associated with antigenic sites (Wanzeck, Boyd, & McCullers, 2011).

She et al. (2017) investigated influenza HA and NA glycosylation of two high‐yield candidate reassortment vaccines (NIBRG‐121xp and NYMC‐X181A) based on the A/California/7/2009 pandemic virus. Nine HA and six NA glycosylation sites were characterized by LC/MS with LTQFT. The authors used a multistage MS/MS approach whereby the peptide + HexNAc (Y1) daughter ion from MS^2^ is targeted in MS^3^ scanning. As previously described, this ion is often the most abundant daughter ion in the glycopeptide MS^2^ spectrum. Targeting Y_1_ in this way allowed abundant peptide ion fragmentation. Therefore, MS^2^ provided high quality glycosyl fragment ion abundances whilst MS^3^ provided for high quality peptidyl fragment ion abundances. The analyses revealed site specific locations of sulfated complex glycans, a substitution that has been linked to increased viral infection in MDCK cell lines (Ichimiya et al., 2014).

Wang et al. (1993) also investigated HA and NA glycan sulfation in several other influenza viruses. Sulfation of glycosylation site N146 in A/Tokyo/3/67 NA has been associated with changes in virulence, enzymatic activity and viral replication (Wang et al., 2009; Li et al., 1993). This work sought to further investigate the extent of sulfation modification in the influenza antigens. Orbitrap MS with CID and HCD were utilized in the study for the collection of glycosyl and peptidyl rich fragment data respectively. The authors used minimal manipulation and neutral pH during sample preparation in an effort to preserve sulfatated glycans. Viruses analyzed including A/Michigan/45/2015(H1N1), A/Switzerland/9715293/2013(H3N2), A/turkey/Turkey/1/2005‐(H5N1), A/Anhui/1/2013(H7N9), and B/Brisbane/60/2008. Sulfation was most often seen in Gal‐GlcNAc (substituting Gal) and GalNAc of the complex N‐glycans. Sulfation was also seen to a lesser extent on GlcNAc of Gal‐GlcNAc motifs extending from the core mannotriose. Based on sulfation patterns the authors inferred sulfotransferase substrate specificities, suggesting that LacNAc extensions are required for GlcNAc substitution with sulfate on a neighboring branch. However, some caution must be used in such interpretation as intramolecular sulfate migration can occur in the gas phase (Thatcher, 1996). Large tetra‐antennary N‐glycans were seen with sulfated LacNAc extensions similar to those seen in keratans (Funderburgh, 2002). Overall the authors reported that, on average, 60% of all glycans were sulfated. Topological analyses of sulfated N‐glycans revealed site specific locations of sulfation on both influenza glycoproteins HA and NA. Both hen egg and MDCK cells were used in this study and each had sulfated glycans localized to overlapping glycosylation sites in both HA and NA proteins. Currently there are no glycosylation patterns reported for either HA or NA produced in infected tissues. Therefore, it is not known if these patterns occur during infection. However, the reported advantage that sulfation provides in virulence, activity and viral replication strongly suggests that these modifications are biologically important (Li et al., 1993; Wang et al., 2009).

It has been long speculated that the cell system used to derive influenza vaccine antigen may affect vaccine function due to differences in glycosylation. Certainly, loss or gain of glycosylation sites has been linked to changes in protection in animal systems (Skowronski et al., 2014; Zost et al., 2017). Batch to batch shifts in glycosylation may also impact vaccines. In addition, it was unclear if the single radial immunodiffusion (SRID) potency assay used for batch release during flu vaccine production is sensitive to changes in glycosylation. An and co‐workers examined the glycosylation patterns on H1N1, H3N2 and type B virus antigens present in the 2014‐2015 influenza vaccines. Specifically, strains A/California/07/2009 H1N1, A/Texas/50/2012 H3N2, and B/Massachusetts/02/2012 grown in egg, MDCK and sf9 cell substrates were examined. MALDI ToF MS was used to analyze released and permethylated glycans. Compositions were assigned using an in‐house program. Glycopeptides were analyzed by LC/MS^E^. An in‐house developed software program, GLYMPS, was used in processing and data validated by manual inspection. Glycans were modeled to HA surfaces based on X‐ray crystal structures available in the NCBI protein database. Insect antigen was expressed as recombinant HA. All others were from whole virus. One hundred and twelve site specific glycan compositions were identified. Overall profiles differed significantly. As expected, insect HA contained mostly paucimannose glycans. H1N1 egg‐derived HA contained more complex glycan than the MDCK derived form. H3N2 and Type‐B were opposite in glycan subtype distributions. Specific glycosylation sites also differed depending on cell type. For example, pandemic A/California/07/2009 H1N1 differed considerably at the head site N87 with MDCK‐derived samples exhibiting nearly all forms as complex whilst egg‐derived was about half complex. Within each HA type (H1, H3, or B), egg‐derived versus MDCK‐derived site‐specific glycan subtypes differed by 5–50% when grouped as high mannose versus complex/hybrid, with a minority of sites that were essentially identical. Each antigen was tested in the SRID assay with and without prior glycan release using a mixture of endo F1, F2, and F3 enzymes, which can be used on proteins in their native state. Glycan release was 70% efficient or greater. Strikingly, the deglycosylation had little effect on the SRID assay results suggesting that it is not sensitive to changes in glycosylation. This study showed significant cell type dependent differences in glycosylation pattern on a glycosite by glycosite basis and showed that the SRID assay may not be sensitive to glycosylation in some circumstances.

### C Glycomics and Glycoproteomics Lessons From Other Viruses

Herpes simplex virus type 1 (HSV‐1) presents an unusual case in that the major envelope glycoprotein contains primarily O‐glycosylation sites rather than N‐glycosylation sites. O‐glycosylation site analysis is more challenging than N‐glycosylation sites both due to a lack of appropriate enzymes and lack of predictable glycosylation sites as is the case for N‐glycosylation. On the basis of the appearance of Pro, Ser and Thr sequence clustering in envelope proteins, Bagdonaite et al. (2015) investigated HSV‐1 envelope proteins for evidence of O‐glycosylation. Using a panel of carbohydrate specific mAbs the authors demonstrated that HEL fibroblasts expressing HSV‐1 expressed core 1, Sialyl T‐antigen, T‐antigen, and truncated Tn antigen O‐glycans. Follow‐up β‐elimination and LTQ‐Orbitrap nanoLC/MS analysis of glycans revealed the presence of Tn (GalNAc only glycosylation of Ser/Thr), core‐1, and low levels of core‐2 O‐glycans. To investigate O‐glycopeptides, trypsinized samples were desialylated and enriched using peanut agglutinin (PNA) and Vicia villosa lectin (VVA). Using an ETD fragmentation strategy, 74 O‐glycosylation sites on 8 envelope proteins were identified. Env protein gB has two positions occupied, T53 and T480, that have been described as necessary for host receptor interaction (37). O‐linked glycans were found throughout the ectodomain and localized to both ordered and unstructured regions of the molecule. Env protein gD contained conserved O‐glycosites at the Ig‐core and flanking regions required for interaction with nectin. Similar structure‐function inferences were made across the env proteins examined in the study. Using lectin staining and ER and Golgi markers, the authors were able to infer that the full secretory repertoire of glycosyltransferases required for O‐glycosylation were present and active even though infection reorganized the secretory stacks. HaCaT cells with COSMC knockout, which produce only Tn and Sialyl Tn O‐glycans, were used to produce HSV‐1. Those with truncated O‐glycans were greatly reduced in infectivity as measured by a Vero cell infection assay. Therefore, elongated O‐glycans are likely required for efficient infection. A weakness of the study was that O‐glycans were desialylated prior to analysis. Sialylation was inferred based on mAb and lectin staining. It may prove interesting to apply selective SA derivatization strategies, which could shed light on SA density and linkage specificity (Yang et al., 2018b; Pongracz, Wuhrer, & de Haan, 2019) in these proteins.

Yang et al. (2018a) developed a chemoenzymatic method for analysis of O‐glycopeptides utilizing a commercially available O‐protease OpeRATOR. This enzyme has broad specificity across most mammalian O‐glycan core types and cleaves peptide bonds at the N‐terminus of glycosylated Ser and Thr residues. The method utilizes an aldehyde resin for linkage of enriched tryptic glycopeptides to solid phase. N‐glycans are first enzymatically released to be analyzed separately. Then the attached glycopeptides are treated with the OpeRATOR enzyme to release O‐glycopeptides leaving the remaining peptides without O‐glycosites on the resin. Using this method, recombinant Zika virus E and NS2 proteins were characterized. Eight O‐glycosites on E and 31 on NS2 were reported.

Parsons et al. (2019) investigated the role of glycosylation in the RBD of infectious bronchitis virus M41 spike protein. The RBD is highly glycosylated with 10 N‐glycosylation sites. Each of the glycosylation sites were knocked out using alanine substitution (N to A) and each mutant form was examined by LC/MS^E^ and the data processed using the in‐house informatics software, GLYMPS. Site occupancy estimates were also calculated. Released glycan analysis was performed by semi‐quantitative permethylation analysis. Circular dichroism with melt curve analysis was used to assay two‐dimensional structural components. Site specific glycosylation was highly similar in most variants. Six of the ten glycosylation variants lost binding to chicken trachea tissue and an Sialylα2,3‐ substituted oligosaccharide ligand. The ligand was chosen based on glycan array analysis of the RBD construct (Wickramasinghe et al., 2011). Released glycans profiles were highly similar except for one variant, which bound more strongly to receptor. Interestingly, the gain of function mutant also had the most dramatic shift in global glycosylation pattern from the wild‐type form with a shift toward more intermediate sized sialylated and fucosylated glycans. Molecular modeling studies suggested a carbohydrate–carbohydrate‐dependent interaction at the putative receptor site within the RBD galectin fold.

Glycomes are not always predictable. Most glycoinformatics software rely upon glycan libraries. Even those that are more discovery based and build saccharides based on monosaccharide components and associated fragments are limited in the glycans for which they can predict based on their a priori make‐up. The ability to determine linkage, anomeric configuration, and the particular diastereomer make‐up of glycans, especially those present on glycopeptides, is limited at best. Therefore, the vast majority of glycomics studies in viruses rely upon inferred structural information, at least in part. While such approaches may be appropriate in many or most cases there is always a danger that the unexpected structural configuration will be missed or misinterpreted. The analyst must always be mindful of the unexpected. Therefore, we cannot completely extricate our more modern analytical approaches from more traditional ones such as those including high field nuclear magnetic resonance (NMR), compositional analysis by chemical means including GC/MS analysis of derivatives, and differential chemical reactivities. By way of example, the last investigation summarized below demonstrates the more traditional approach needed when encountering an undefined system.

De Castro et al. (2013) investigated glycosylation of the major capsid protein Vp54 from the prototype chlorovirus *Paramecium bursaria* chlorella virus 1 (PBCV‐1). The virus is large and encodes a set of glycosyltransferases and other glyco‐synthetic enzymes. The major capsid protein Vp54 contains post translational modifications approximating 6 kDa based on protein sequence. The accepted eukaryotic N‐glycosylation sequon is not present. Early reports found the presence of seven neutral monosaccharides including, glucose, fucose, galactose, mannose, xylose, rhamnose, and arabinose (Wang et al., 1993). Monosaccharide and linkage analysis were performed by GC/MS analysis of partially methylated alditol acetates. A range of high field NMR experiments were performed at the glycopetide level to determine fine structure. Single and multiple heteronuclear bond correlations were determined by heteronuclear single quantum coherence and heteronuclear multiple quantum coherence, respectively. Scalar coupled systems were mapped by TOCSY. Long range couplings were removed and further connectivities were revealed by TROESY. MALDI ToF/ToF revealed peptide sequence and glycan heterogeneity. De minimus and de maximis configurations were revealed. Key features included the presence of both D and L forms of Rha of which, in the former, methylation of C2 and C3 positions of terminal forms were reported. The glycosylation sequons were unique and 4 of the 5 were in the NTXT and the fifth in the NIPG contexts. This work reported a previously unknown glycosylation pathway that exists outside of the three major domains of life.

## VIII REVEALING THE HOST SIDE OF VIRAL GLYCOMICS

This section will discuss glycomics characterization of host tissues targeted by viral pathogens, cell substrates used in virus propagation and glycan arrays used to analyze viral receptor specificity for glycan ligands. It should be noted that early glycan arrays were largely constructed from synthetic glycans. More recently, arrays have been constructed from glycans released from natural sources. Glycomics strategies used to characterize natural glycans use in arrays will be briefly reviewed. Lastly, key studies using arrays that have revealed important properties of viral interaction with saccharide receptors will be discussed.

There has been increased interest in the glycan profiles of tissues targeted by viruses. Walther et al. (2013) to examine human respiratory tissue. This study was performed particularly to examine whether or not lung oligosaccahrides were represented in the synthetic glycan arrays available at the time up to 2013. Lung and bronchial tissue N‐ and O‐glyans were analyzed. MALDI‐ToF and MALDI ToF/ToF analyses of permethylated glycans were used for compositional and fragmentation analyses, respectively. GC/MS analysis of partially methylated alditol acetates was used to provide monosaccharide identities and linkage configurations. This approach has been used extensively in glycomics analysis and has been reviewed (North et al., 2010). Glycans approaching 6000 amu were detected many of which contained extended poly LacNAc (Galβ1,4GlcNAcβ1,3)*_n_* antennary extensions of up to six repeats per antennae. Antennae were capped with both NeuAcα2,3 and NeuAcα2,6. These were the main type of complex glycan found in both tissue lung and bronchus showed different replication rates when a range of avian and seasonal influenza were cultured with explants. On the basis of the glycomics identifications and replication rates seen in *ex vivo* replication experiments 32 glycans were examined in array format. The results showed that most viruses preferred poly LacNAc configurations. This was one of the first studies to correlate tissue tropism with glycan structure and glycan array technology.

A nearly identical approach was utilized to investigate the ferret glycome. The ferret is a premier model system to study influenza Jia et al. (2014) reported the glycolipids, N‐ and O‐glycomes of ferret respiratory tissues. Lung and trachea were studied. The approach employed MALDI‐ToF analysis of permethylated glycans, fragmentation using MALDI‐ToF/ToF MS, and GC/MS analysis of partially methylated alditol acetates to provide monosaccharide identities and linkage configurations. MALDI‐ToF was performed in reflection positive mode. As found in human respiratory tissues, high molecular weight N‐glycans were observed consistent with many structures containing extended antennae composed of poly LacNAc terminated with SA. A minority of N‐glycans contained the Sda epitope, which describes the GalNAcβ1,4(NeuAcα2,3)GlcNAc‐ configuration.

The use of glycan arrays to asses viral binding to different host glycoforms has been highly informative. The technical aspects of current glycan arrays have been recently reviewed (Oyelaran & Gildersleeve, 2009; Geissner & Seeberger, 2016) and will not be covered in depth in this section. Rather, some technical aspects relevant to the studies, what they have potentially revealed, the current directions of their development and current state of mass spectrometry based analytics used in their construction and characterization will be discussed particularly for native glycan arrays. A wide range of human viral pathogens recognize glycans as at least part of their receptor specificity and have also been recently reviewed (Thompson, de Vries, & Paulson, 2019). The viruses most analyzed in this way are influenza, parainfluenza, and coronavirus. The value of these arrays will be discussed for these three viruses.

A wealth of information concerning influenza receptor specificities has been generated with the advent of glycan array technology. About a decade ago it was widely held that avian viruses primarily recognized sialylα2,3‐linked glycans, human influenza recognized sialylα2,6 and swine viruses recognized both forms of substitutions (Gamblin & Skehel, 2010). As synthetic arrays printed with synthetic, well‐defined glycans came into use, it became clear that not only the SA linkage, but also the structural attributes of the underlying glycan are important (Ji et al., 2017; Stencel‐Baerenwald et al., 2014). Many of these studies were performed with the Center for Disease Control (CDC) or Consortium for Functional Glycomics (CFG) (Amonsen et al., 2007; Bradley et al., 2011; Tappert, Smith, & Air, 2011; Gulati et al., 2013, 2014; Walther et al., 2013; Jernigan & Cox, 2015; Pappas et al., 2015; Pulit‐Penaloza et al., 2017; Hosoda et al., 2018; Byrd‐Leotis et al., 2019a) arrays. Detection of binding on arrays has been through direct labeling with fluorophore or through secondary detection with labeled antibody. Both whole virus and recombinant influenza HA has been studied by array. A clear pattern has emerged that shows a higher preference of avian and pandemic strains for Sialylα2,3‐substituted glycans with their close human seasonal flu relatives preferring Sialylα2,6‐substituted glycans (Gulati et al., 2014; Stevens et al., 2006a,b; Liao et al., 2010). Binding preferences can change dynamically as the virus passages through the human populations. With use of arrays, de Vries and co‐workers (de Vries et al., 2011) demonstrated that in some cases only two receptor site residues need be changed to switch ligand preference. Closely related seasonal and pandemic H1 HAs were studied. Two amino acids in HAs, T200 and E227, were found to significantly shift preference toward Sialylα2,6 glycans. In another study, fifty‐three type B influenza strains were analyzed (Wang et al., 2012) revealing three categories (1) those that recognized primarily sialyl‐α2,6; (2) those that recognized both Sialylα2,3 and Sialylα2,6; and (3) those that primarily recognized sulfated glycans. These grouped along the lines of classifications used in choice of vaccine strains as Yamagata‐like, and the latter two Victoria‐like. The importance of sulfated host ligands is gaining in recognition (Ichimiya et al., 2014). Other studies utilizing glycan arrays have shown that glycosylation sites added in the receptor site domain can facilitate ligand preference changes (Jayaraman et al., 2012; Zhu et al., 2015). Glycan arrays have also been used to track avian strains for potential to make the zoonotic leap to human (Zhu et al., 2015) which may provide information leading to better predictability of pandemics.

The majority of influenza studies have been performed with glycan arrays printed with synthetically derived glycans and a minority that are highly purified from natural sources. An interesting outcome of some studies was the preferential recognition of short poly‐LacNAc repeat glycans as inferred from previous discussion through the glycomics study of human and ferret respiratory tissue. Later versions of the CDC and CFG arrays have short LacNAc repeat glycans of 2–3 units. Recent arrays have incorporated longer poly‐LacNAc compounds (Peng et al., 2017) and analyses with them suggest that preference for poly‐LacNAc glycans in seasonal influenza A virus has not changed in 40 years (Yang et al., 2015; Alymova et al., 2016) and may have arisen as consequence of added glycosylation sites to the HA head (Yang et al., 2015). It should be noted that, without glycomics study of viral target tissue such discoveries would not have been possible. These poly LacNAcs more closely mimic glycans found in host tissues including ferrets (Jia et al., 2014) and humans (Walther et al., 2013). The current model for interaction between HA and sialylated poly‐LacNAc predicts that long poly‐LacNAc glycans engage in a bidentate interaction across HA multimer subunits (Ji et al., 2017). Synthetic arrays, host tissue glycomics, and careful examination of the evidence have revealed preferred glycan ligands and preferences across influenza strains. However, what is being missed in synthetic arrays? The answer to this question is emerging through mass spectrometry driven glycomics study of tissue glycans that are subsequently arrayed.

Natural glycan arrays, also known as shotgun arrays, have been constructed from host tissues including swine (Byrd‐Leotis et al., 2014) and human (Byrd‐Leotis et al., 2019a). If collected in sufficient quantities, fractions containing glycans of interest can be revisited and analyzed in more depth. In efforts to facilitate such pathways, Song and co‐worker developed a hypochlorite releasing method reported to release from large sample sizes to yield gram quantities of glycan material that include N‐glycan, O‐glycan, and glycolipids (Irungu et al., 2007) and this method was used in the human and swine glycan array construction. The glycans are separated by two‐dimensional chromatography and fractions analyzed by MALDI ToF/ToF MS (Song et al., 2011a, 2014). The data are collected in positive ion mode where little linkage or branch information is revealed. A lectin panel is used to further characterize arrayed glycans, yielding inferred monosaccharide and some linkage information. While the arrays may contain previously unidentified natural glycans, the glycans are not as completely characterized as synthetic glycans, leaving ambiguity in binding data. A recent human array study found evidence that some HAs binds another class of glycans: phosphorylated mannans normally seen in the targeting of proteins to lysosomes (Byrd‐Leotis et al., 2019b). While mass spectrometry analyses combined with lectin characterization of the arrayed natural glycans did not yield fine structural detail, identification f such compounds can be informative. Using a similar approach, a shotgun glycan array was produced using pig lung released glycolipid, N‐ and O‐glycans (ref). Influenza containing H1, H2, H3, and H6 HAs were examined, which run the range of avian, human and swine viruses. As expected, avian strains bound most strongly to Sialylα2,3 glycans. Human derived forms bound most strongly to Sialylα2,6 glycans, and swine derived viruses bound to both types of sialyl linkage. Key glycans most informative on the array were further interrogated through MALDI‐ToF/ToF fragmentation analysis in both positive and negative polarity modes. Notably absent from the array, but detected in tissues, were poly LacNAc N‐glycans. However, they were present in tissues but not in sufficient amounts for inclusion in the array. The authors did note that many influenza strains bound to poly LacNAc glycans on synthetic arrays. The work on both human and swine shotgun arrays demonstrated that if sufficient amount of natural glycans are available it is possible to perform follow‐up characterization on glycans of interest to reveal sufficient structural detail to address biological questions.

In efforts to facilitate better up‐front analytics a number of groups are developing informatics approaches to facilitate glycan array assignments (Aoki‐Kinoshita, 2018; Jankowska et al., 2018; Grant et al., 2016; Kletter et al., 2015; Venkataraman, Sasisekharan, & Raman, 2015; Agravat, Saltz, Cummings, & Smith, 2014; Cholleti et al., 2012). Overall, these software allow visualization of data sets, perform first pass glycan assignments and collate lectin and mass spectrometric data to facilitate structural interpretation. As natural arrays advance so must the analytical platforms used in characterization especially in situations where large sample sizes are not feasible.

Parainfluenza virus causes respiratory illness in humans. There are four major serotypes, which include HPIV‐1, HPIV‐2, HPIV‐3, and HPIV‐4; the first three cause the majority of human illness. Unlike influenza where HA and NA are two separate proteins, in HPIV both activities are on the same protein, HA–NA. There is a balance between the two activities that governs infection, progression of infection through cell‐cell fusion, and entry into the lytic cycle (Moscona & Peluso, 1992). Differences in the activities of the NA across strains tends to impact these attributes where higher activity leads to faster clearance of sialyl receptors, less cell‐cell contact, and reduced infection resulting in a more likely lower grade infection or entry into the lytic cycle. Glycan arrays have been used to answer questions regarding the HN protein such as: how do the ligands/targets of H and N differ (Tappert, Smith, & Air, 2011), does a conformational change govern exposure and activity of active sites of N (Mahon et al., 2008), and are there more than one binding site on NA? (Mishin et al., 2010).

HPIV‐1 and HPIV‐3 were studied by CFG V2 glycan array containing 285 synthetic glycans (Amonsen et al., 2007). While these arrays largely contain synthetic glycans, it should be noted that, their inclusion as compounds relevant to biomedical processes is supported by a large volume of glycomics data present in the literature. Viruses were fluorescently labeled with Alexa 488. Both HPIV‐1 and HPIV‐3 bound to oligosaccharides containing NeuAc‐α2‐3Gal‐β1‐4GlcNAc, including sialyl‐Lewis x. Sulfated glycans were also good ligands. The pH and temperature set points were used to study binding and digestion parameters presumably by the H and N components of HNs. HN associated with poly‐LacNAc glycans even in the absence of sulfate or Fucose. HPIV‐3 bound sulfated glycans whereas HPIV‐1 did not. Both viruses had a preference for NeuAc‐α2,3. The HPIV1‐3 virus was further studied using multiple CFG glycan arrays including v. 2.0 (4), v. 3.2 (406 glycans), 4.0 (442 glycans), 4.1 (465 glycans), and/or 4.2 (511 glycans) at both pH 5 and pH 7 to separate H and N activities, the latter being more active at pH 5 (Tappert, Smith, & Air, 2011). The inhibitor 2‐deoxy‐2,3‐didehydro‐N‐acetylneuraminic acid (DANA) was used to block NA activity. On the basis of comparisons of glycan array results to those using red blood cells and fetuin as ligands, the authors reported that protein conformation and cellular context likely has an impact on H binding and susceptibilities to N digestion. Arrayed glycans with ~0.5 nm separation were more susceptible than those displayed on protein or cell surfaces. These distances were reported to likely allow multiple binding sites on the HN multimer NA to be engaged. Previous studies reported that at lower pH range the HN protein undergoes a conformational change exposing the N active site to facilitate NA activity. The array studies support this notion (Crennell et al., 2000). The HPIV viruses have broader N specificity than H specificity if the substrate is fixed to a surface, consistent with a surface‐induced conformational change in HN. Loss of an N‐glycosylation site on HPIV‐1 HN site 173 unveiled an additional receptor site, site 2, active in both ligand binding and NA activity (Mishin et al., 2010). The mutant was isolated based on its insensitivity to inhibitor BCX 2798, an inhibitor of site 1. Glycan array analysis (CFG v3.2; 406 glycans), in similar strategies as above and with the inhibitor, was used to characterize binding specificity of both sites. Sites 1 and 2 had much ligand cross over. However, unlike site 1, site 2 was found to have specificity for NeuAcα2,8‐substituted glycans. Mutants containing the mutation unveiling site 2, N173S, has been isolated from the clinic (Henrickson & Savatski, 1992).

Coronavirus are pathogens of humans and avian species, causing a significant impact on domestic fowl. The avian form, coronavirus γ, infects respiratory and several non‐respiratory tissues, such as the gastrointestinal, oviduct, and kidney. The RBD of the host recognition moiety of the spike protein has been studied in some detail. Glycoconjugates appear to be the dominant ligands and likely differ across tissues, defining tropism of these viruses. The tropism of a range of strains has been studied using tissue slides. Treatment with glycosidases has confirmed that, in the case of Infectious bronchitis virus (IBV), SA is required for binding of the spike protein RBD to tissues. The ligand range appears to be far narrower than for IAV (Wickramasinghe et al., 2011). Avian strain M41, studied with glycan array using CDC versions 4.1 and 4.2 revealed binding to compounds containing Neu5Acα2,3 Gal with most intense binding to Neu5Acα2,3 Galα1,3(Neu5Acα2,3 Galα1,4)GlcNAc. The M41 virus was studied using tissue arrays from a range of fowl. Using glycan as inhibitor, the tissue arrays suggested that M41 preferred Neu5Acα2,3 Galα1,3 over Neu5Acα2,3 Galα1,4. Using CFG glycan array v 5.1, which contained both compounds, this observation was borne out (Ambepitiya Wickramasinghe et al., 2015a). In a study of pigeon and partridge coronavirus (CoV) spike protein (S1), the S1 proteins did not bind any of the 610 glycans on the array although NA destroyed binding to tissue arrays indicating S1 binds some form of SA (Bouwman et al., 2020). One possible reason for this discrepancy is the absence of the specific ligand of pigeon and partridge S1 on the array. There are currently no natural glycan arrays for these hosts. Binding of enterotropic avian gamma coronaviruses, including turkey coronavirus (TCoV), guineafowl coronavirus (GfCoV), and quail coronavirus (QCoV), which are evolutionarily distant from respiratory avian coronaviruses based on the viral attachment protein spike (S1) have been studied by glycan array (Ambepitiya Wickramasinghe et al., 2015b). Tissue arrays showed that TCoV, GfCoV, and QCoV aligned with their particular tissue tropisms. Glycan array analyses revealed that, in contrast to the S1 protein of IBV M41, S1 proteins of these enteric viruses recognize nonsialylated poly‐N‐acetyl‐lactosamines. In the case of the gamma coronaviruses, tissue and glycan arrays have paired well to, at least in part, decode the carbohydrate dependent infectious process. A clear next step would be to perform detailed analysis of these tissue's glycomes to reveal any possible natural ligands, which would then be arrayed for further study. Another possibility would be to perform MALDI ToF MS imaging experiments to further localize ligands within the tissues (Holst et al., 2016; Drake et al., 2018).

Bovine coronavirus (BCoV) has also been investigated by glycan array using CFG v 5.0 printed with 611 glycans. The spike protein has been crystalized revealing a galectin fold similar to that of human galectins. Neu5,9Ac2 was found to have the highest response on glycan array. Using X‐ray structure information and mutagenesis studies, critical residues required for interaction with Neu5,9Ac2 were identified. Middle East Respiratory Syndrome coronavirus (MERS‐CoV), a respiratory pathogen of humans and camels, has also been studied by glycan array (version of slide not provided). Chromeo488‐conjugated secondary StrepMAB antibody was used for detection. In addition to binding to a cell surface entry receptor, the virus also binds carbohydrate ligand via its spike protein subdomain S1^A^. Glycan array analysis revealed a preference for α2,3‐linked over α2,6‐linked SAs. Interestingly, 5‐N‐glycolylation and (7,)9‐O‐acetylation hampered binding. On the basis of their studies, the authors speculated that high and low affinity receptor sites govern interaction during early viral attachment. Spike protein domain A predominantly bound to short, sulfated, Sialylα2,3‐linked mono‐sialotrisaccharides and to long, branched, Sialylα2,3‐ substituted diantennary and triantennary glycans with a minimum extension of 3 Galβ1,4GlcNAc‐β1,3 (LacNAc) tandem repeats. Inclusion of uncommon SAs was inspired by previous glycomics studies (Butor, Diaz, & Varki, 1993; Shi, Chammas, & Varki, 1996). For the array three different glycans were modified using a three enzyme preparation including *Escherichia coli* sialicacid aldolase, *Neisseria meningitidis* CMP‐SA synthetase and *Photobacteriumdamselae* α2–6‐sialyltransferase to produce naturally occurring rare SAs (Yu et al., 2006). Glycans were characterized by MALDI‐ToF, MALDI‐TOF/TOF and a panel of biotinylated lectins (Song et al., 2011b). Again, glycomics study renders array technology leading to informed biomedical analysis.

## IX SYNOPSIS

Mass spectrometry advances have paralleled advances in viral glycomics analysis. Multiple fragmentation modes have been partnered to reveal key aspects of viral structure–function relationships and pairing these modes together such as CID/HCD and HCD/ETD have enhanced spectral information content leading to better structurally defining fragmentation patters for both peptidyl and glycosyl moieties of glycopeptides. More recent advances such as SWATH and MS^E^ have increased coverage and accuracy of quantitation in the latter over a much less limited data acquisition compared to more standard DDA acquisitions. The recent introduction of UVPD fragmentation in some way mirrors FT‐ICR IRMPD and may be tunable for targeted glycomics applications. Highly productive protease strategies have been introduced to yield improved glycosite coverage and multi‐polarity mode approaches have also contributed toward improved glycosite coverage. Sialic acid derivatization methods are now available to differentiate and quantitate SA linkages in free glycans and glycopeptides. Unique endoglycosidase and N‐peptidyl glycosidase strategies have been introduced to reveal key aspects of glycosite glycan subtypes for inferred regiospecific function while simplifying the inherent heterogeneity. New glycosidases and O‐proteases are being introduced facilitating better characterization of O‐glycans and O‐glycopeptides.

These and other advances have facilitated a better understanding of key viral glycoproteins, their functions, roles in vaccine development, and in host–pathogen interactions. Differences between glycosylation patterns of monomeric and multimeric protein states have been unveiled. Antigenic masking has been demonstrated in numerous cases through mapping of glycosylation states to antigenic sites. High mannose regions on HIV‐1 envelope proteins have been well characterized, revealing that key host antibodies target the high mannose glycan patch impacting vaccine design. Assigning glycan subtypes regiospecifically to the peptide backbone has also driven the study of important immune system lectins and collectins and the influence viral glycosylation has on virus removal and the immune response. Glycan arrays have revealed much information regarding preferred receptor ligands and the dynamics of preference changes over time as viruses traverse host populations. Overall, the co‐evolution of mass spectrometry technology and interest in viral glycomics have advanced both areas.

## X FUTURE DIRECTIONS

Glycomics and mass spectrometry have made tremendous strides in recent years. Advances in several crucial areas could facilitate another leap in analytical abilities, further facilitating a greater understanding of structure–function relationships in viral glycomics. Glycoinformatics, for example, remains an area in need of advancement. In part, this is a limitation imposed by the information content available in glycomics data. Polarity mode, fragmentation mechanism, adduct type, and derivatization type all have bearing on the characteristics of fragment ions observed and their abundances. In terms of derivatization, while some strategies have great advantages, for example permethylation of glycans, such methods are not always acceptable such as when native forms are required for downstream processes or when substitutions are labile or replaced during derivatization. Therefore, it is necessary to develop alternative chemistries and better informatics for multiple modes of data collection. In terms of free glycans for analysis, there is a growing list of spectral libraries being developed or already available (Everest‐Dass et al., 2013; Campbell et al., 2014; Toghi Eshghi et al., 2015; Xue, Laine, & Matta, 2015; Pai, Hu, & Lam, 2016; Ashline, Zhang, & Reinhold, 2017; Remoroza et al., 2018; Ashwood et al., 2019; Ye et al., 2019). Informatics driven by such libraries should improve accuracy of assignments if appropriate standards, cross‐lab validation, cross‐instrument validation, and library curation is employed. Current glycopeptide analysis is limited to compositional assignment of the glycolyl portion with caveats even for derived sequence information (Wuhrer, Deelder, & van der Burgt, 2011). Better methods need to be developed for deeper structural analysis of these compounds. Finally, separation technologies are not currently adequate to resolve complex samples with released glycans or glycopeptides. Ion mobility is a relatively new technique, which adds a level of separation in the gas phase. Ion mobility resolving power is currently low in most commercial instruments but improved instrument designs, such as cyclic ion mobility, promise to extend this range considerably (Giles et al., 2019; Ujma et al., 2019).

## ABBREVIATIONS

ER: endoplasmic reticulum
Fuc: fucose
Gal: galactose
GalNAc: *N*‐acetylgalactosamine
GalNAc‐T: GalNAc transferase
Glc: glucose
GlcA: glucuronic acid
GlcNAc: *N*‐acetylglucosamine
Hex: hexose
HexNAc: hexosamine
Man: mannose
NeuAc: sialic acid
PTM: post‐translational modification
Xyl: xylose
